# On two new species of deep-sea carrier crabs (Crustacea, Brachyura, Homolodromiidae, *Dicranodromia*) from Taiwan and the Philippines, with notes on other Indo-West Pacific species

**DOI:** 10.3897/zookeys.1072.72978

**Published:** 2021-11-22

**Authors:** Peter K. L. Ng, Chien-Hui Yang

**Affiliations:** 1 Lee Kong Chian Natural History Museum, National University of Singapore, 2 Conservatory Drive, Singapore 117377, Republic of Singapore National University of Singapore Singapore Singapore; 2 National Taiwan Ocean University, 2 Pei-Ning Road, Keelung 20224, Taiwan National Taiwan Ocean University Keelung Taiwan

**Keywords:** Comparative taxonomy, deep-sea crab, East Asia, Homolodromioidea, systematics.

## Abstract

The systematics of four species of the homolodromiid genus *Dicranodromia* A. Milne-Edwards, 1880, from East Asia and the Philippines is reappraised: *D.danielae* Ng & McLay, 2005, *D.doederleini* Ortmann, 1892, *D.karubar* Guinot, 1993, and *D.martini* Guinot, 1995; and key characters such as the epistome, gonopods, and spermatheca are figured in detail. Two new species, *D.erinaceus***sp. nov.** and *D.robusta***sp. nov.**, are described from Taiwan and the Philippines, respectively. *Dicranodromiaerinaceus***sp. nov.** resembles *D.spinulata* Guinot, 1995, and *D.delli* Ahyong, 2008 (from New Caledonia and New Zealand) but can be separated by its distinctly spinulated carapace surfaces and proportionately shorter fifth ambulatory legs. *Dicranodromiarobusta***sp. nov.** is superficially similar to *D.baffini* (Alcock & Anderson, 1899) and *D.karubar* Guinot, 1993, but can easily be separated by possessing a broad dorsoventrally flattened infraorbital tooth. A genetic study of the species using the mitochondrial cytochrome c oxidase I gene confirms that the taxa are distinct, with *D.erinaceus***sp. nov.** coming out in a well-supported clade from congeners. The megalopa of *D.doederleini* is also reported for the first time.

## Introduction

The deep-water carrier crabs of the homolodromiid genus *Dicranodromia* A. Milne-Edwards, 1880, are represented by 20 species from the Atlantic, Indian, and Pacific Oceans (Guinot 1995; Ng and McLay 2005; Ng and Naruse 2007; Ahyong 2008; Ng et al. 2008; Tavares and Lemaitre 2014). Of these, 11 species are known from the Indo-West Pacific: *D.baffini* (Alcock & Anderson, 1899), *D.chenae* Ng & Naruse, 2007, *D.crosnieri* Guinot, 1995, *D.danielae* Ng & McLay, 2005, *D.delli* Ahyong, 2008, *D.doederleini* Ortmann, 1892, *D.foersteri* Guinot, 1993, *D.karubar* Guinot, 1993, *D.martini* Guinot, 1995, *D.nagaii* Guinot, 1995, and *D.spinulata* Guinot, 1995.

We describe two additional species from Taiwan and the Philippines. The Taiwanese material had been misidentified as “*D.doederleini*” by earlier workers, while the Philippine specimens had been incorrectly identified by field collectors as “*D.delli*”. We also take this opportunity to update the character states of some poorly known species and refigure them so that they are better defined. In particular, we add male first and second gonopod characters for the species as they are useful to discriminate some of the taxa. Their taxonomy is also discussed. In addition, we also report on the larvae of an ovigerous female of *D.doederleini* which had been kept in the aquarium.

## Materials and methods

Material examined is deposited in the National Taiwan Ocean University (**NTOU**), Keelung, Taiwan; and the Zoological Reference Collection (**ZRC**) of the Lee Kong Chian Natural History Museum, National University of Singapore. Measurements are provided in millimetres of the maximum carapace width and length. The ambulatory leg articles are measured along their maximum length while the width is determined at midlength where it is widest.

The terminology used follows Guinot (1995) and Davie et al. (2015). In *Dicranodromia*, the groove of G1 in which the G2 is inserted is on the dorsal surface of the structure (relative to the carapace), and in situ, the lobes present on the subdistal part are on the outer margin, i.e., directed laterally outwards. The following abbreviations are used: coll. = collected by; G1 = male first gonopod; G2 = male second gonopod; P2–P5 = pereiopods 2–5 (ambulatory legs 1–4).

For the molecular analysis, a total of seven individuals were used (as indicated on the material examined of each species below). Two species, *D.danielae* and *D.robusta* sp. nov., could not be tested as they had originally been preserved in formalin. Crude genomic DNA was extracted from the muscles of the pleon using QIAamp DNA Micro Kit (Qiagen, Cat. No. 56304, Valencia, CA, USA) following the protocol of the manufacturer. The DNA barcoding gene (mitochondrial cytochrome c oxidase I, COI; cf. Hebert et al. 2005) was amplified using the universal primer set (LCO1490/HCO2198, 657 bp; Folmer et al. 1994). PCR reaction components, temperature cycling conditions and sequencing reaction followed those used in Ng et al. (2018). For comparisons, the COI sequence of *Homolodromiakai* Guinot, 1993 (voucher number ZRC 2018.0109) was obtained and used as the outgroup for the analysis.

The resulting sequences were firstly translated into the corresponding amino acids by EditSeq (LASERGENE; DNASTAR) to check for pseudogenes (Song et al. 2008). BioEdit v.7.1.3.0 (Hall 1999) was then used to align and edit the COI dataset, with MEGA v. 7 (Kumar et al. 2016) used to calculate the uncorrected pairwise distances (*p*-distance). Maximum likelihood (ML) method was used to construct the phylogenetic tree by RAxML v.7.2.6 (Stamatakis 2006). ML analysis settings followed the model of general time reversible with a gamma distribution GTRGAMMA) for the COI dataset. Branch confidence of the tree topology was assessed using 1,000 bootstrap replicates (Felsenstein 1985).

## Systematic account

### Family Homolodromiidae Alcock, 1899

#### Genus Dicranodromia

Taxon classificationAnimaliaDecapodaHomolodromiidae

A. Milne-Edwards, 1880

A009AEED-883B-519F-AC65-41037B373165

##### Type species.

*Dicranodromiaovata* A. Milne-Edwards, 1880, by monotypy; gender feminine).

##### Remarks.

As the number of species has increased and more material has been examined since the revision by Guinot (1995), a revised key to *Dicranodromia* is provided below for the Indo-West Pacific species. To date, there are no shared species between the Atlantic and Indo-West Pacific.

#### Key to Indo-West Pacific species of *Dicranodromia*

**Table:** 

1	Basal antennal article relatively short, stout; anteroexternal tooth long, subequal to or longer than rest of article	**2**
–	Basal antennal article more elongate; anteroexternal tooth short, distinctly shorter than rest of article	**7**
2	Carapace and pereiopods covered with short and very dense plumose setae, forming velvet-like tomentum, obscuring surfaces and margins of pereiopods	**3**
–	Setae of various types and lengths on carapace and pereiopods, can be relatively dense but never obscuring surfaces and margins of pereiopods	**4**
3	Carapace proportionately wider, anterolateral and posterolateral margins unarmed or only with small granules [southern Java and Moluccas]	***D.karubar* Guinot, 1993**
–	Carapace proportionately narrower, anterolateral and posterolateral margins lined with sharp spinules or granules [Indian Ocean]	***D.baffini* (Alcock & Anderson, 1899)**
4	Infraorbital tooth broad, dentiform to linguiform, subequal or larger than exorbital tooth [Philippines]	***D.robusta* sp. nov.**
–	Infraorbital tooth triangular, smaller than exorbital tooth	**5**
5	Anterior surface of epistome prominently spinose; P2 and P3 merus with distinct spines on flexor margin [Philippines]	***D.danielae* Ng & McLay, 2005**
–	Anterior surface of epistome at most granulate or with scattered spinules; flexor margin of P2 and P3 merus unarmed	**6**
6	P2 and P3 dactyli short, propodus more than twice length of dactylus [Philippines]	***D.chenae* Ng & Naruse, 2007**
–	P2 and P3 dactyli long, propodus 1.5–1.6× length of dactylus carapace [Philippines]	***D.martini* Guinot, 1995**
7	Dorsal surface of carapace covered with distinct spinules, especially along lateral parts, those on median parts may be present as granules; flexor margin of P2–P5 merus distinctly lined with spines	**8**
–	Dorsal surface of the carapace almost smooth, with granules or spinules present only on lateral parts; P2 and P3 merus unarmed except for flexor margin sometimes with spinules, P4 and P5 merus unarmed	**11**
8	Exorbital tooth exorbital tooth triangular, dentiform; posterior margin of epistome prominently spinose [Madagascar]	***D.crosnieri* Guinot, 1995**
–	Exorbital tooth slender, spiniform; posterior margin of epistome entire, adjacent area smooth or with scattered spinules	**9**
9	Median part of dorsal surface of carapace covered with distinct spinules; submarginal area of posterior margin of epistome with several spinules; P2 and P3 relatively short (e.g., P3 propodus less than 7× longer than wide; propodus 1.7× length of dactylus) [New Caledonia; New Zealand]	***D.spinulata* Guinot, 1995**
–	Median part of dorsal surface of carapace covered with granules, not spinules; submarginal area of posterior margin of epistome unarmed; P2 and P3 relatively longer (e.g., P3 propodus more than 8× longer than wide; propodus 1.7× length of dactylus)	**10**
10	P2–P5 proportionately shorter (e.g., P3 merus 4.5× longer than wide; P5 merus just reaching branchiocardiac groove when folded dorsally) [Taiwan]	***D.erinaceus* sp. nov.**
–	P2–P5 proportionately longer (e.g., P3 merus 6.6× longer than wide; P5 merus longer and more slender, extending beyond branchiocardiac groove when folded dorsally) [New Zealand]	***D.delli* Ahyong, 2008**
11	Posterior margin of epistome entire; outer surface of palm in both sexes evenly covered with granules [Chesterfield Islands]	***D.foersteri* Guinot, 1993**
–	Posterior margin of epistome crenulate; median outer surface of palm in both sexes smooth, granules only present on subdorsal and subventral margins	**12**
12	Carapace and pereiopods covered with numerous long stiff setae but not obscuring surfaces and margins [known for certain only from Japan]	***D.doederleini* Ortmann, 1892**
–	Carapace and pereiopods densely covered with numerous setae of different types; partially obscuring carapace surface and margins, but almost completely obscuring surfaces and margins of pereiopods [Japan]	***D.nagaii* Guinot, 1995**


#### Dicranodromia doederleini

Taxon classificationAnimaliaDecapodaHomolodromiidae

Ortmann, 1892

2DD6BCCC-FC80-5E3F-B1A9-B3CF2831CB59

Figures 1 , 2 , 3

Dicranodromia doederleini Ortmann, 1892: 549, pl. 26, fig. 4; Guinot 1995: 202, figs 2C, 11a, c, d, 12A–C; Ikeda 1998: 54–55, pl. 1 figs 1–6; Ng et al. 2008: 39 (for complete synonymy, see Guinot 1995: 202).

##### Material examined.

Japan: 1 ♀ with 1 megalops (15.9 × 20.2 mm), Sagami Bay, from aquarium trade, 8 Apr. 2015 (ZRC 2017.1214, COI sequence: OK351331); 1 ovigerous ♀ (14.5 × 19.2 mm), 35°32.51'N, 139°54.74'E, Futttsu, Kanaya, Chiba Prefecture, 200–250 m, 19 Sep. 2007 (ZRC 2021.0469, COI sequence: OK351333); 1♂ (10.3 × 8.5 mm), station 29, 34°40.21'N, 139°18.62'E, SW of Izu-Ohshima Island, Izu Islands, 289-307 m, TRV Shin’yo-maru, 2002 research cruise, coll. T. Komai, 24 Oct. 2002 (CBM-ZC 16572, COI sequence: OK351332).

##### Remarks.

This species is well known (for synonymy and records, see Guinot 1995; Ikeda 1998) but may be a species complex, and specimens from outside the type locality in Japan all need to be rechecked (see Guinot 1995; Ng and McLay 2005).

**Figure 1.**
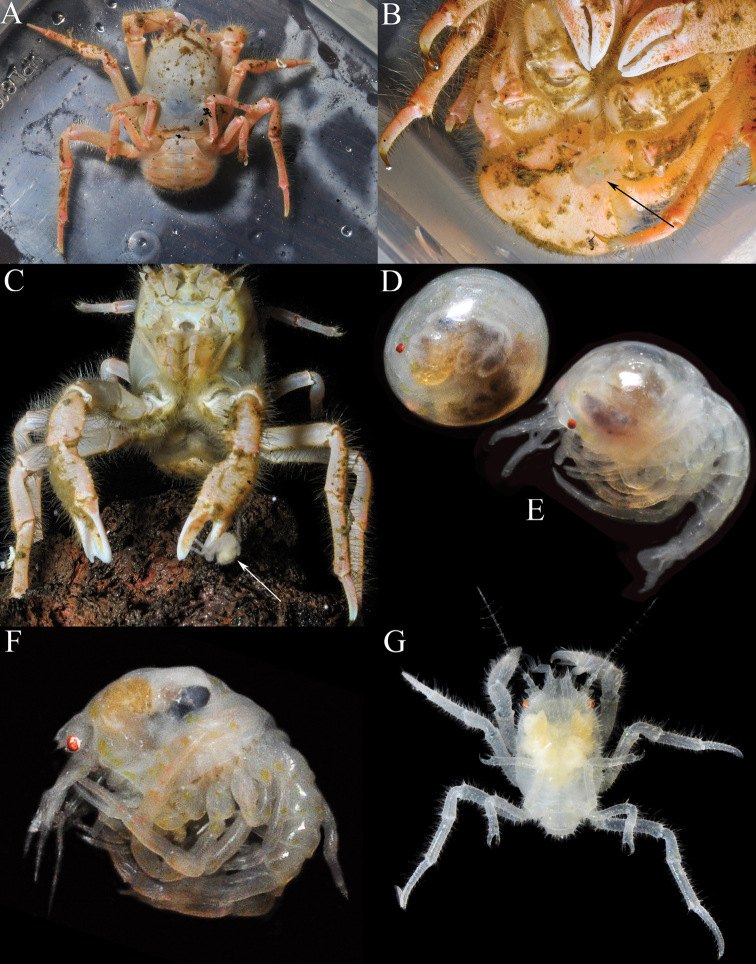
*Dicranodromiadoederleini* Ortmann, 1892, ♀ (15.9 × 20.2 mm) (ZRC 2017.1214), Japan **A** colour in life (26 April 2015) **B** ventral surface showing megalopa (arrow) (26 April 2015) **C** frontal view showing megalopa crawling on chela (arrow) and larvae under pleon (24 April 2015) **D** fresh eyed egg (not preserved, 18 April 2015) **E** first zoea (not preserved, 18 April 2015) **F** freshly hatched megalopa (not preserved, 18 April 2015) **G** dorsal view of free moving megalops (26 April 2015). Photographs: PYC Ng.

One female specimen (ZRC 2017.1214) was imported to Singapore via the aquarium trade in early April 2015. On 8 April, the specimen was obtained by Paul YC Ng and observed to have between 10–20 large eggs under the pleon with the eyes just visible. It was kept in a cold-water aquarium (ca. 15–20°C) with other crustacean and fish species. On 18 April, he noted that several eggs had been released into the aquarium (Fig. 1D) which appeared ready to hatch, and that some of the egg membranes had ruptured revealing what appeared to be a dead first zoeal stage (Fig. 1E). One specimen, however, was apparently a freshly hatched and dead megalopa (Fig. 1F). He observed the first free-crawling megalopa on the female specimen on 24 April (Fig. 1B, G). Unfortunately, no larvae except one megalopa was preserved (PYC Ng, personal communication).

**Figure 2.**
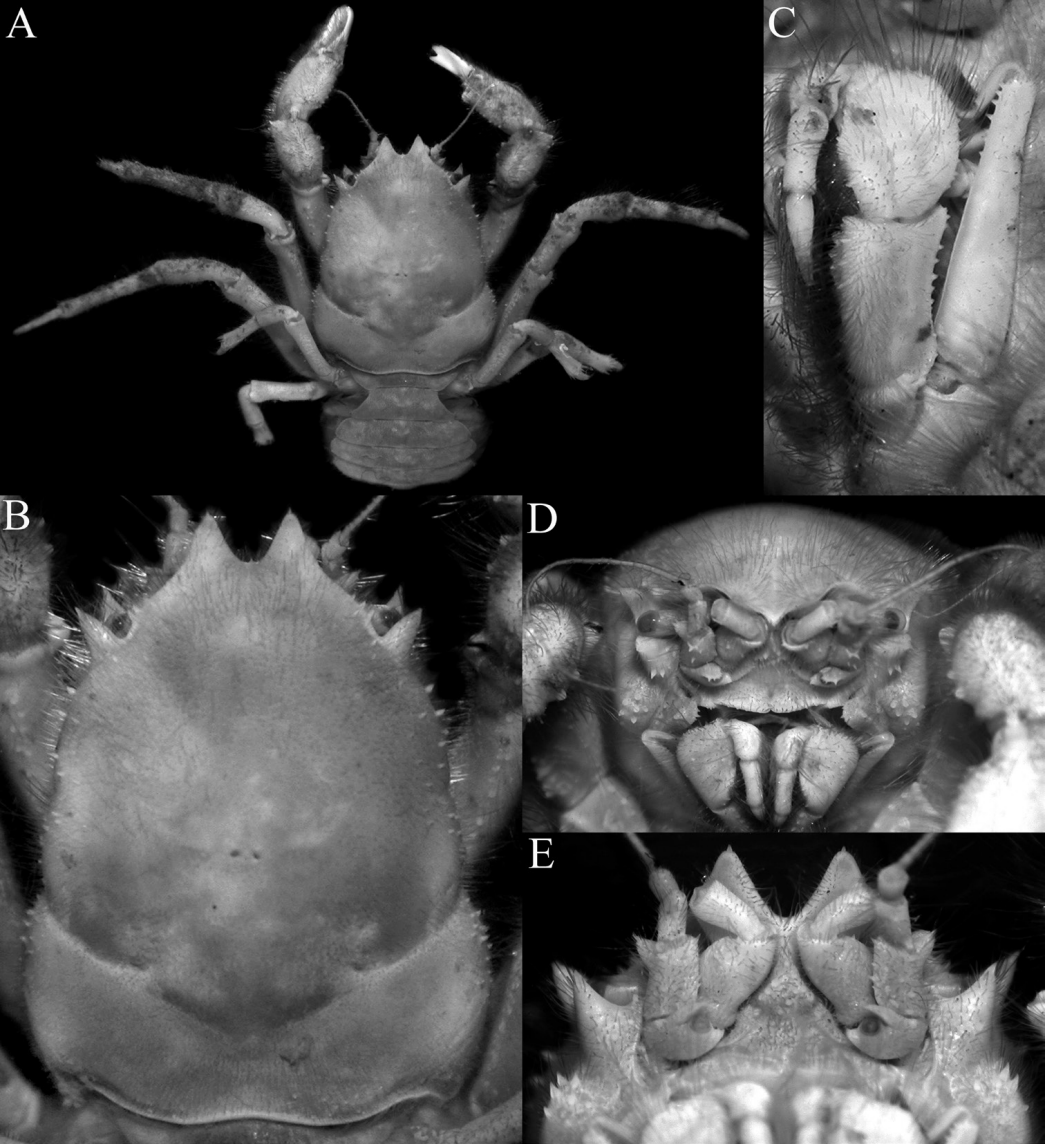
*Dicranodromiadoederleini* Ortmann, 1892, ovigerous ♀ (14.5 × 19.2 mm) (ZRC 2021.0469), Japan **A** overall view **B** dorsal view of carapace **C** left third maxilliped **D** frontal view of cephalothorax **E** epistome, antennules, antennae and orbits.

**Figure 3.**
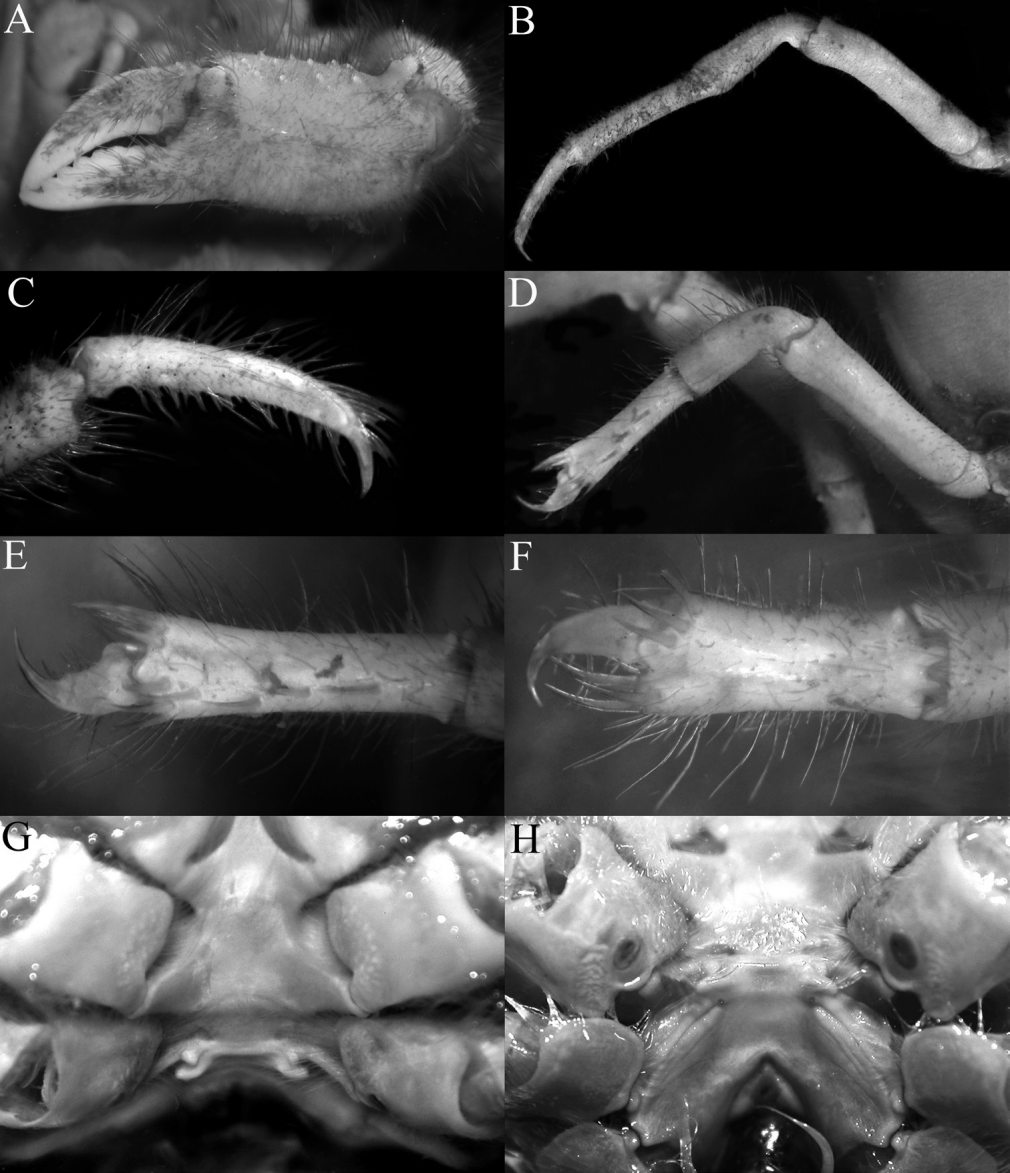
*Dicranodromiadoederleini* Ortmann, 1892, ovigerous ♀ (14.5 × 19.2 mm) (ZRC 2021.0469), Japan **A** left chela **B** left P3 **C** right P3 dactylus **D** left P5 **E** left P5 propodus and dactylus **F** left P4 propodus and dactylus **G** anterior thoracic sternum and spermatheca **H** posterior thoracic sternum showing spermatheca and female gonopores.

The observations above on the eggs and megalopa of *D.doederleini* provide some clarity on the larval development in the genus. While it is known the eggs are large and the development is abbreviated, it is not sure of the eggs hatch into an advanced zoeal stage or directly into megalopa. Caustier (1895) was the first to report on the first zoea of *Dicranodromiaovata* A. Milne-Edwards, 1880, from the Atlantic but he based this on unhatched embryos and unfortunately, the description was brief, and no figures were provided. Martin (1991) found a specimen of *D.felderi* Martin, 1990 from the western Atlantic, which had well-developed eggs and removed some embryos. On the basis of these, he described what he regarded was the first zoea. Guinot (1995: 105) reported that a specimen of *D.nagaii* from Japan had about 20 megalopae under the pleon and suggested the eggs hatched directly into this stage. The eggs of *D.doederleini* are full of yolk, and even the “first zoea” observed are of the lecitotrophic type, with yolk sacs and appendages, which are poorly or not setose (Fig. 1E). They are very similar to the condition observed or the dromiid *Cryptodromiapileifera* Alcock, 1900 which has only one lecitrophic first zoeal stage before the megalopa (Tan et al. 1986). In *Cryptodromiapileifera*, however, the zoea is still able to swim and move around in the water column although it only lasts two days before metamorphosing. For the specimen of *D.doederleini* that was kept in the aquarium, it would appear that if it was natural, the young would develop into an advanced zoeal stage while still inside the egg membrane, and break free only after it metamorphoses into the megalopa. The transition between the “first zoea” and megalopa, however, is clearly very short, perhaps a day or less. The condition for *Dicranodromia* is thus probably similar to that of eubrachyuran marine crabs, some other podotreme crabs and various enbrachyurans like the epialtids *Paranaxiaserpulifera* (Guérin, 1832) and *P.keesingi* Hosie & Hara, 2016 (Rathbun 1914, 1924; Morgan 1987; Hosie and Hara 2016), and the pilumnids *Pilumnusnovaezealandiae* Filhol, 1885 and *P.lumpinus* Bennett, 1964 (cf. Wear 1967); taxa which undergo direct development.

#### Dicranodromia martini

Taxon classificationAnimaliaDecapodaHomolodromiidae

Guinot, 1995

F760E67A-909E-5938-AE37-AF4516C6608B

**Figure 4.**
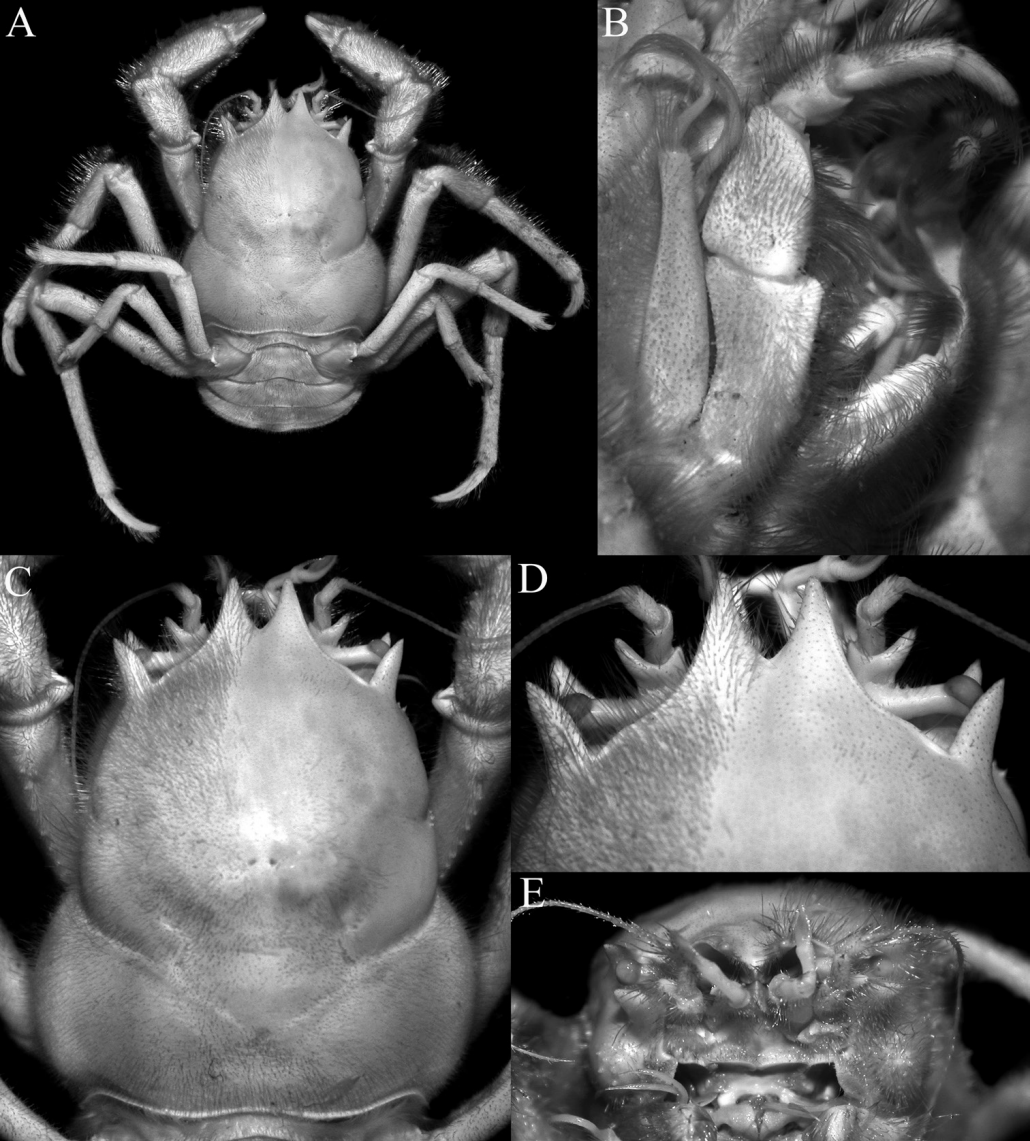
*Dicranodromiamartini* Guinot, 1995, ♀ (28.1 v 34.2 mm) (ZRC 2007.0106), Philippines **A** overall view **B** right third maxilliped **C** dorsal view of carapace **D** anterior part of carapace (right side denuded) **E** frontal view of cephalothorax.

**Figure 5.**
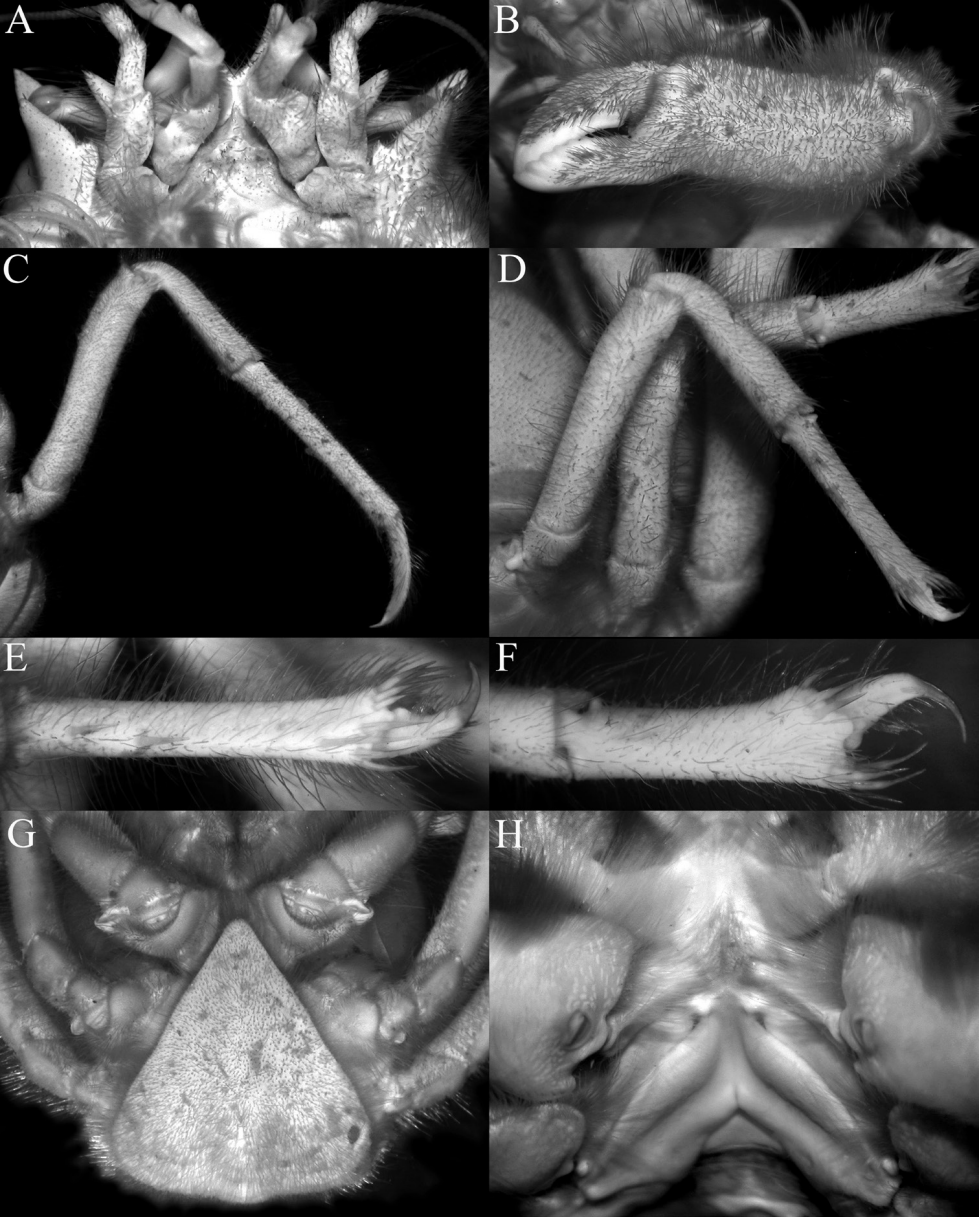
*Dicranodromiamartini* Guinot, 1995, ♀ (28.1 × 34.2 mm) (ZRC 2007.0106), Philippines **A** epistome, antennules, antennae and orbits **B** left chela **C** right P3 **D** right P5 **E** right P5 dactylus **F** right P4 **G** telson **H** posterior thoracic sternum showing spermatheca and female gonopores.

**Figure 6.**
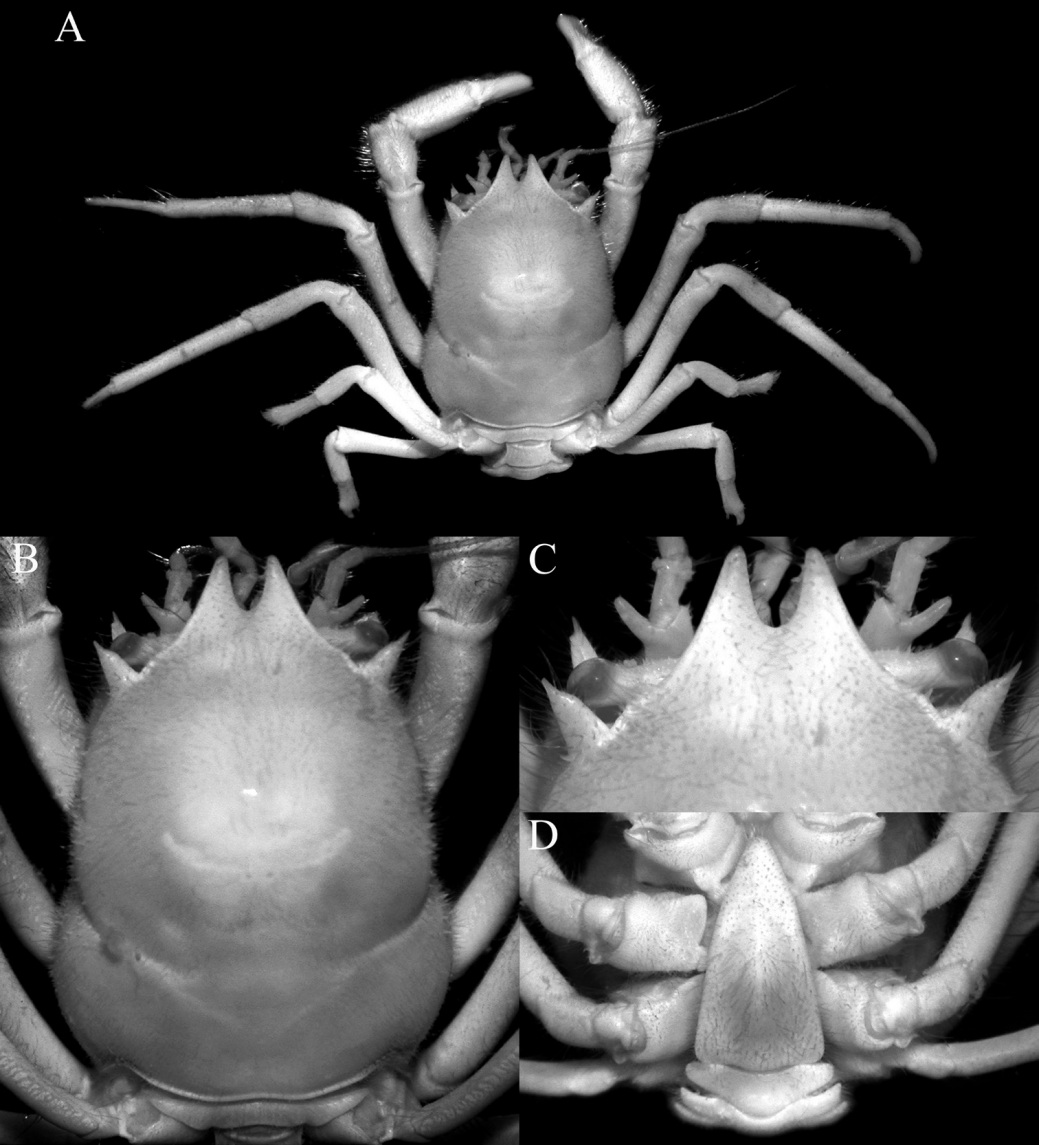
*Dicranodromiamartini* Guinot, 1995, ♂ (12.3 × 16.6 mm) (ZRC 2007.0105), Philippines **A** overall view **B** dorsal view of carapace **C** anterior part of carapace (partially denuded) **D** male telson.

Figures 4 , 5 , 6 , 11A–C

Dicranodromia martini Guinot, 1995: 221, figs 19a–e, 20A–C; Ng and Naruse 2007: 48, figs 1, 3a, b, 4; Ng et al. 2008: 39, fig. 11.

##### Material examined.

Philippines: l ♂ (12.3 × 16.6 mm), station CP2396, 9°36.3'N, 123°42.0'E, Maribohoc Bay, Panglao, Bohol, Visayas, 609–673 m, PANGLAO 2005 Expedition, coll. MV DA-BFAR, 31 May 2005 (ZRC 2007.0105); l ♀ (28.1 × 34.2 mm), station CP2363, 9°06.0'N, 123°25.0'E, Bohol and Sulu Seas, 437–439 m, PANGLAO 2005 Expedition, coll. MV DA-BFAR, 26 May 2005 (ZRC 2007.0106, COI sequence: OK331337).

##### Remarks.

Ng and Naruse (2007: 49) commented that the largest female they examined (28.1 × 34.2 mm, ZRC 2007.0106) has the carapace relatively more inflated with the posterolateral margin distinctly convex and the external orbital tooth more anteriorly directed when compared to smaller males. In addition, this female specimen is also relatively more hirsute (Fig. 4A, C, D versus Fig. 6A–C). We see a similar pattern of variation in *D.erinaceus* sp. nov., where the smaller males are less swollen and with less setae overall when compared to larger females (Fig. 16A, B versus Fig. 13A, B). In *D.karubar*, the exorbital tooth varies in the angle its directed outwards (Figs 8B, C, 10B). As such, the differences observed for the specimens of *D.martini* examined here are regarded as intraspecific and/or size related.

**Figure 7.**
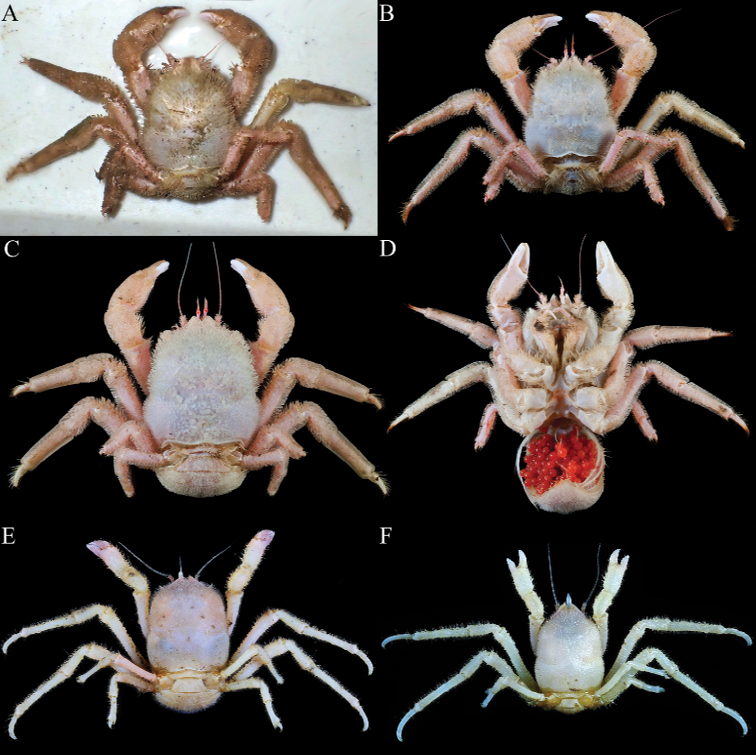
Colour in life. **A,B***Dicranodromiakarubar* Guinot, 1993, ♂ (28.7 × 34.7 mm) (ZRC 2020.0348), Java **C***D.karubar* Guinot, 1993, ovigerous ♀ (30.1 × 35.5 mm) (ZRC 2020.0349), Java **D***D.karubar* Guinot, 1993, ovigerous ♀ (24.8 × 31.5 mm) (ZRC 2020.0348), Java **E***D.erinaceus* sp. nov., holotype ♀ (14.0 × 18.0 mm) (NTOU B00126), Taiwan **F***D.erinaceus* sp. nov., paratype ♂ (6.9 × 9.5 mm) (ZRC 2021.0084), Taiwan. Photographs: T.-Y. Chan.

#### Dicranodromia karubar

Taxon classificationAnimaliaDecapodaHomolodromiidae

Guinot, 1993

1E5392D8-EC9A-514B-87B4-8A1806EBAC99

**Figure 8.**
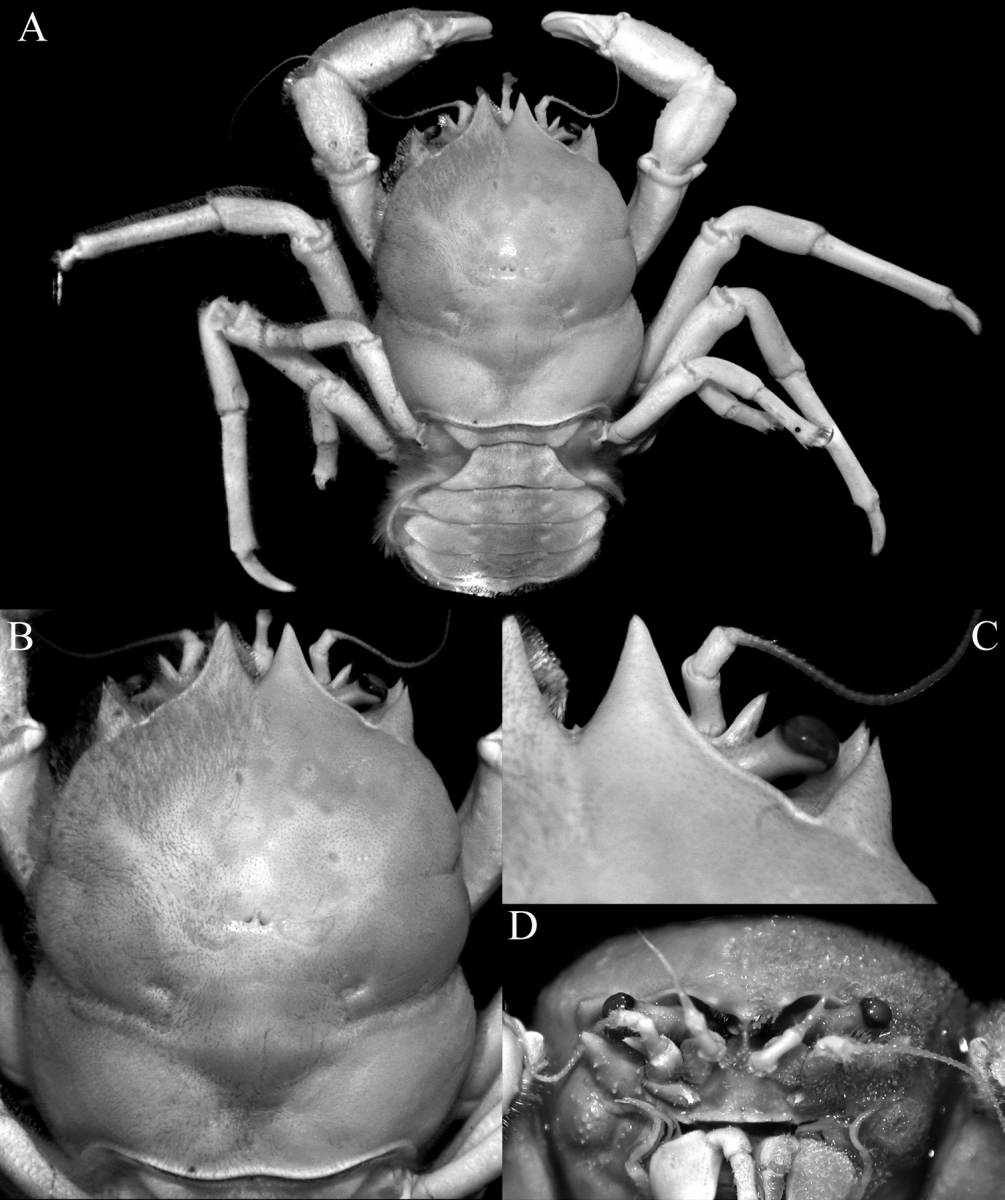
*Dicranodromiakarubar* Guinot, 1993, ♀ (27.1 × 33.4 mm) (ZRC 2020.0348), Java **A** overall view **B** dorsal view of carapace **C** anterior right part of carapace (denuded) **D** frontal view of cephalothorax.

**Figure 9.**
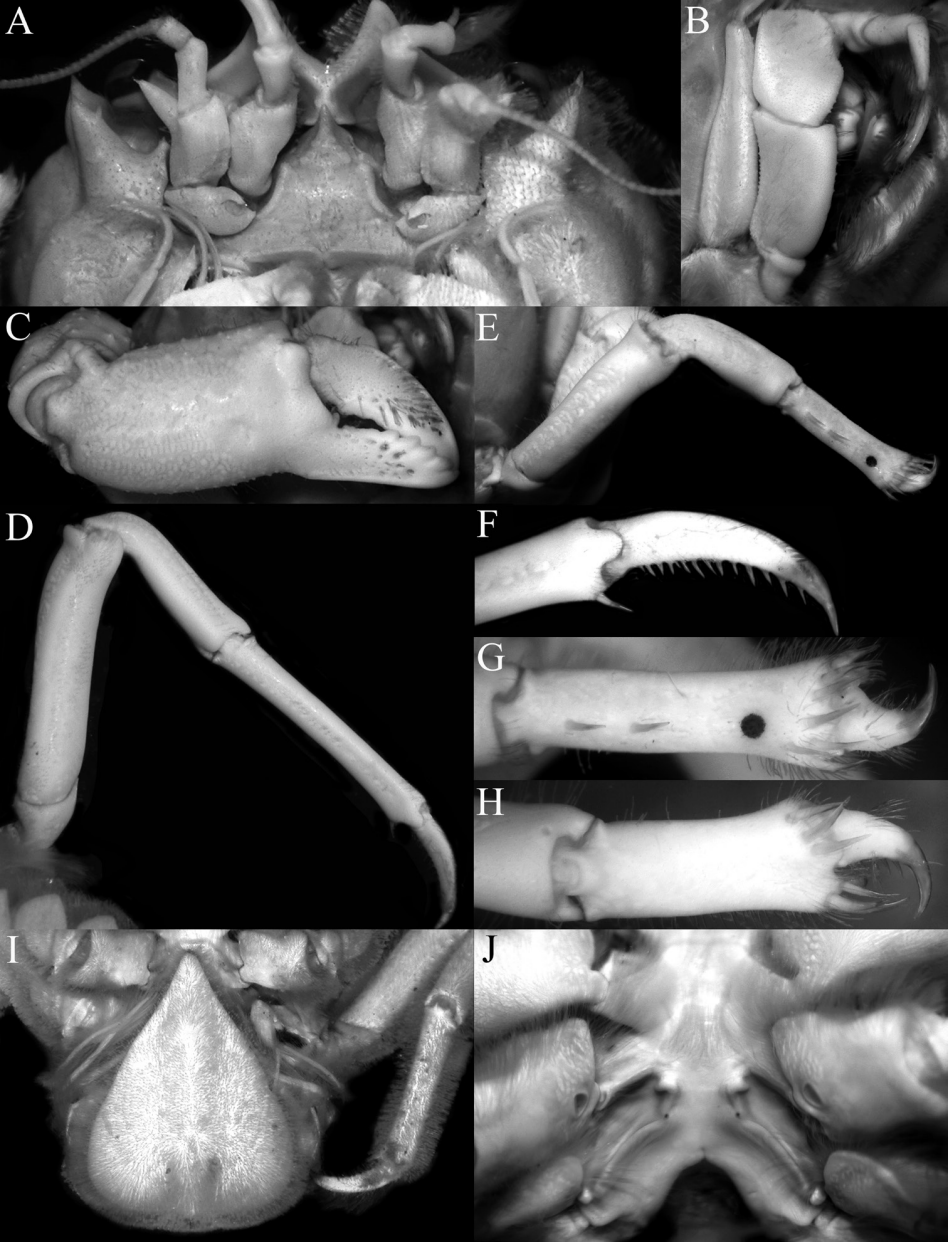
*Dicranodromiakarubar* Guinot, 1993. **A–H, J** ♀ (27.1 × 33.4 mm) (ZRC 2020.0348), Java **I** ♀ (27.6 × 33.8 mm) (ZRC 2020.0348), Java **A** epistome, antennules, antennae and orbits **B** left third maxilliped **C** right chela (denuded) **D** right P3 (denuded) **E** right P3 dactylus (denuded) **F** right P5 (denuded) **G** right P5 propodus and dactylus (partially denuded) **H** right P4 propodus and dactylus (partially denuded) **I** telson **J** posterior thoracic sternum showing spermatheca and female gonopores.

**Figure 10.**
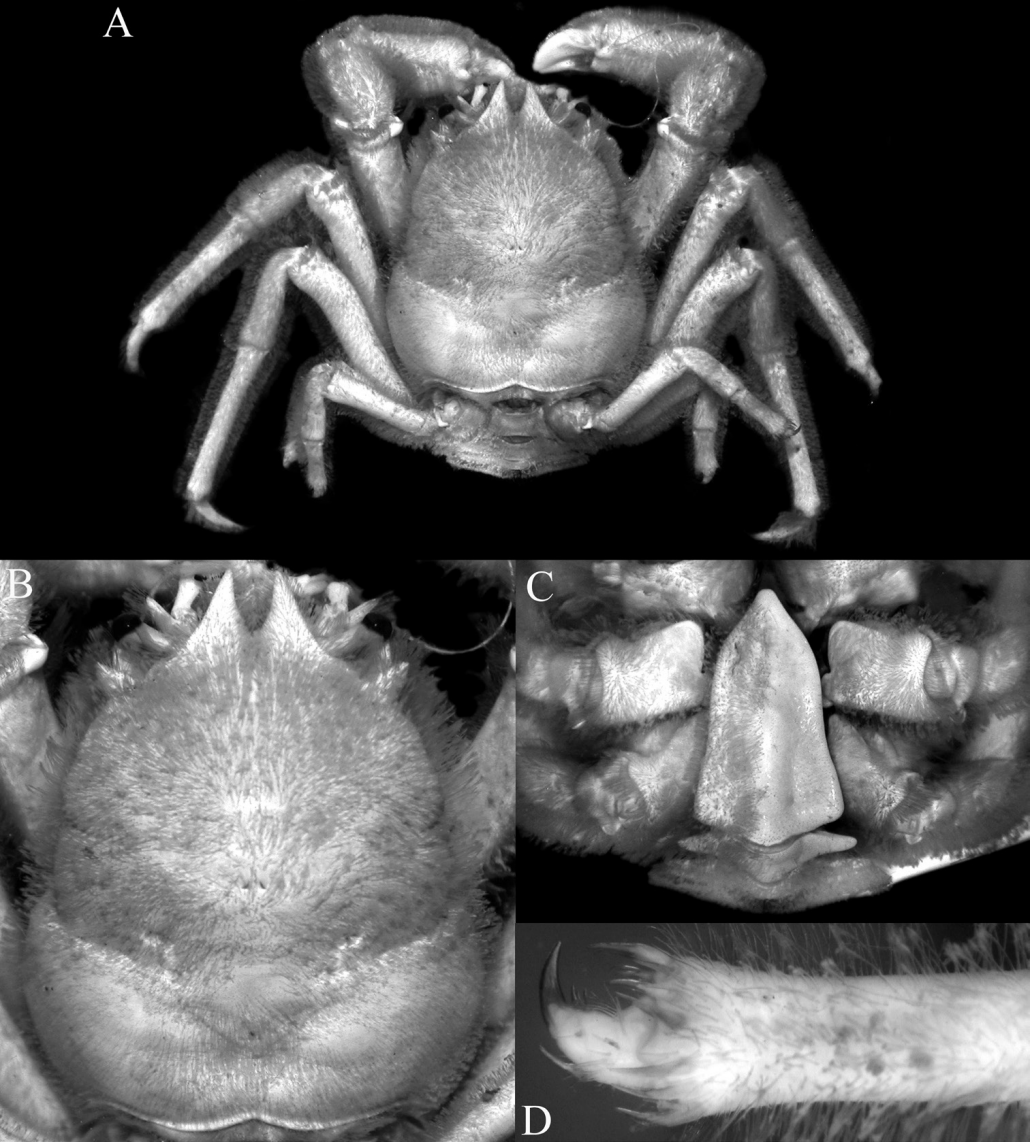
*Dicranodromiakarubar* Guinot, 1993. **A–C** ♂ (28.7 × 34.7 mm) (ZRC 2020.0348), Java **D** ♀ (27.6 × 33.8 mm) (ZRC 2020.0348), Java **A** overall view **B** dorsal view of carapace **C** telson **D** left P5 propodus and dactylus.

**Figure 11.**
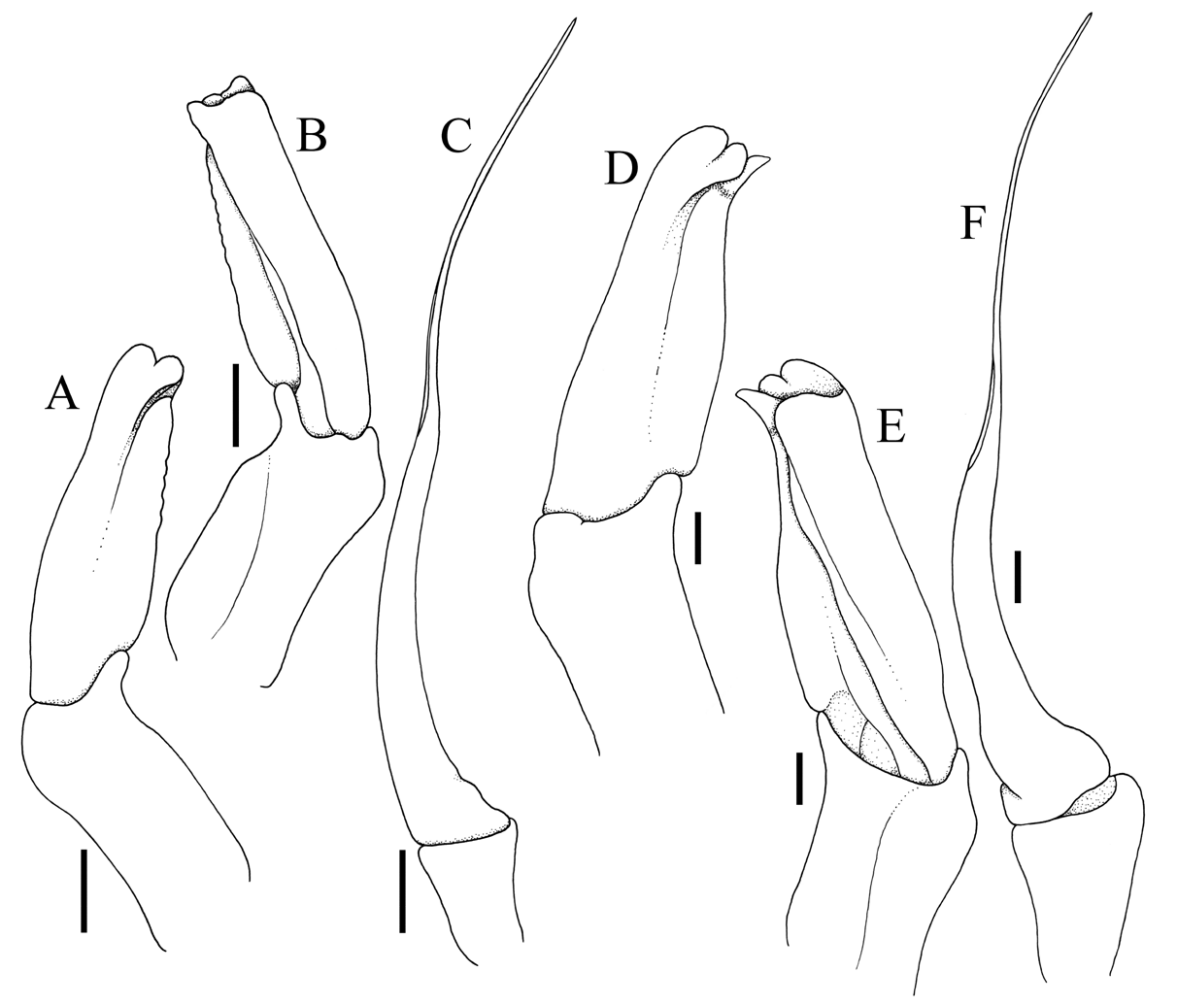
**A–C***Dicranodromiamartini* Guinot, 1995, ♂ (12.3 × 16.6 mm) (ZRC 2007.0105), Philippines **D–F***D.karubar* Guinot, 1993, ♂ (28.7 × 34.7 mm) (ZRC 2020.0348), Java **A,D** left G1 (ventral view) **B,E** left G1 (dorsal view) **C,F** left G2. Setae for all structures not figured. Scale bars: 1.0 mm.

Figures 8 , 9 , 10 , 11D–F

Dicranodromia karubar Guinot, 1993: 213, figs 15A–C, 16A–D, 25A, B; Ng et al. 2008: 39; Mendoza et al. 2021: 284, fig. 1A, B.

##### Material examined.

Indonesia: 1 ♂ (28.7 × 34.7 mm), 3 ovigerous ♀♀ (24.8 × 31.5 mm, 27.1 × 33.4 mm, 27.6 × 33.8 mm), station CP39, 8°15.885'S, 109°10.163'E – 8°16.060'S, 109°10.944'E, 528–637 m, substrate partially muddy, plenty of glass sponges, echinoderms, polychaeta, galatheids, fishes, sea anemone, gastropods and bivalves, south of Cilacap, south Java, Indian Ocean, South Java Deep Sea cruise, coll. beam trawl, 30 Mar. 2020 (ZRC 2020.0348); 1 ovigerous ♀ (30.1 × 35.5 mm), station CP51, 7°04.874'S, 106°25.396'E – 7°05.348'S, 106°25.044'E, 569–657 m, substrate coarse sand, mud and some plastic trash, small crabs, ophiuroids, stalk crinoids, chitons, limpets and sea daisies on fallen bamboo, Pelabuhanratu Bay, south Java, Indian Ocean, South Java Deep Sea cruise, coll. beam trawl, 2 Apr. 2020 (ZRC 2020.0349, COI sequence: OK331336).

##### Remarks.

Mendoza et al. (2021) recently recorded *D.karubar* from southern Java, over 1000 km from its type locality in the Moluccas. The specimens, however, agree very well with the descriptions and figures of Guinot (1995) and they are clearly conspecific.

Guinot (1995: 215) noted that the rostrum of this species is at most a tubercle, which is in conformity with the present material. The merus, carpus and dactylus were described as unarmed by Guinot (1995), but the P5 propodus actually has one or two spines on the outer surface, which are hard to see as the dense plumose setae obscure them. In some specimens, the P5 dactylus has a prominent spine on the extensor margin (Fig. 10D), but as reported by Ng and Naruse (2007), it is absent in others (Fig. 9G, H). The form of the exorbital tooth varies to some degree. In the female specimens, the tooth is clearly directed anteriorly (Fig. 8B, C) but in the male, it is pointed obliquely laterally (Fig. 10B).

The setae on *D.karubar* are unusual in that they are plumose at the distal part (Guinot 1995: fig. 16D). When the animals are freshly collected, the setae lock together to form a dense coat, which traps fine mud and completely obscure the carapace and pereiopod surfaces and margins (Fig. 7A). After the specimen is cleaned gently with a brush and the sediment removed, the surfaces and margins become more visible with the distal plumose parts no longer meshed together. The margins of the pereiopods, however, are still partially obscured as the setae there are denser (Fig. 7B, C). In the form of the setae, *D.karubar* is most similar to *D.baffini* from the Indian Ocean, although the tomentum of the latter species is relatively less dense (cf. Padate et al. 2020: fig. 2a).

*Dicranodromiakarubar* can easily be separated from *D.baffini* by its proportionately broader carapace (Figs 8B, 10B) (versus relatively narrower and longer in *D.baffini*; cf. Guinot 1995: fig. 13, Padate et al. 2020: fig. 2a); the antero- and posterolateral margins almost smooth, except sometimes for a few scattered granules (Figs 8B, 10B) (versus lined with granules and spinules in *D.baffini*; cf. Guinot 1995: fig. 13, Padate et al. 2020: fig. 2a); and the subdistal lobe on the outer margin of the endopod curved and beak-like (Fig. 11D, E) (versus lobe rounded in *D.baffini*; cf. Padate et al. 2020: fig. 2g, i). Based on the figures of Gordon (1950), Guinot (1995: fig. 16C) commented that the structure of the spermatheca was different in the two species, but we do not discern any major differences since both species possess an unusual and prominent comma-shaped tubercle on each side of sternite 7, with the spermatheca at the base of this tubercle (Fig. 9J). The spermathecal structure in the *D.baffini* from the Andamans (cf. Padate et al. 2020: fig. 2j) is almost identical to the condition observed in *D.karubar* (Fig. 9J). Alcock and Anderson (1899: 8) described the structure as “sternal grooves of the female end, without tubercles, at the level of the openings of the oviducts”, which does not match the description and figures of Gordon (1950: 205, text-fig. 1) and Padate et al. (2020: fig. 2j). As noted by Padate et al. (2020: 3), the condition observed by Alcock and Anderson (1899) may be because their specimen was a juvenile.

*Dicranodromiakarubar* is known thus far only from the Moluccas and eastern part of the Indian Ocean while *D.baffini* has been recorded from western India, Maldives and Andamans (Alcock 1899, 1901; Gordon 1950; Padate et al. 2020).

All the females of *D.karubar* collected from the south Javan cruise were ovigerous, the eggs being bright red in life, in a prominent brood pouch (Fig. 7D). One female specimen (27.6 × 33.8 mm, ZRC 2020.0348) had 362 eggs, each about 2.5 mm in diameter.

#### Dicranodromia danielae

Taxon classificationAnimaliaDecapodaHomolodromiidae

Ng & McLay, 2005

111027E9-0561-55B8-BCCC-E1B87C2DEB16

Figure 12

Dicranodromia danielae Ng & McLay, 2005: 40, figs 1–4; Ng and Naruse 2007: 47, fig. 3c; Ng et al. 2008: 39.

##### Material examined.

Philippines: holotype ovigerous ♀ (broken, 10.8 × 14.2 mm), Balicasag Island, Panglao, Bohol, Visayas, in tangle nets, ca. 200–300 m, coll. local shell fishermen, 2 Mar. 2004 (ZRC 2005.0094).

##### Remarks.

**Figure 12.**
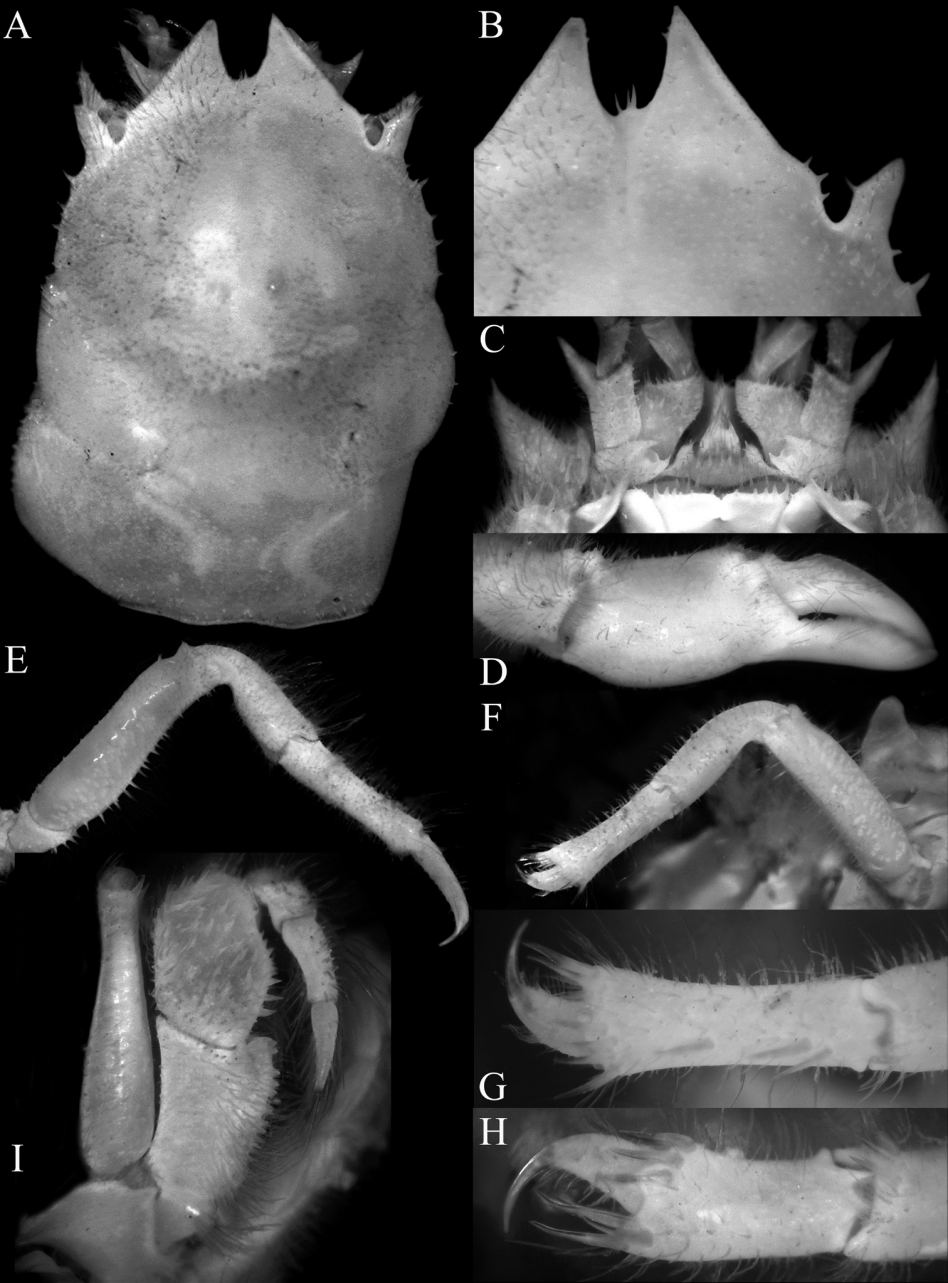
*Dicranodromiadanielae* Ng & McLay, 2005, holotype ovigerous ♀ (broken, 10.8 × 14.2 mm) (ZRC 2005.0094), Philippines **A** dorsal view of carapace **B** frontal region showing rostrum **C** epistome, antennules, antennae and orbits **D** right chela **E** right P3 **F** left P5 **G** left P5 propodus and dactylus **H** left P4 propodus and dactylus **I** right third maxilliped.

The broken holotype female was re-examined and some characters need to be added or amended from Ng and McLay (2005). Ng and Naruse (2007: fig. 3c) had already noted that the P5 dactylus has a distinct spine on the extensor margin (Fig. 12F, G); but in addition, the P5 propodus has three spines on the outer surface (Fig. 12G). The P2 and P3 meri were described being unarmed but this is not correct. The extensor margin has low spines while the flexor margin has a row of slender spines partially covered by the dense stiff setae (Fig. 12D). In addition, the basal antennal article is relatively short with the anteroexternal tooth long and subequal in length to the article (Fig. 12C). In addition, the epistome of this species is unusual in that the distal part is strongly spinose, with the median lateral part possessing a sharp anteriorly directed tooth; and the rostrum consists of two lateral and one median slender spinules (Fig. 12B, C). The merus of the third maxilliped is distinctive, being strongly spinose, with the inner margin lined with strong spines; the exopod is essentially unarmed (Fig. 12I).

Some of the characters of *D.danielae* resemble the male specimen 9.7 × 14.0 mm from Uraga Strait in Japan (35°4.833'N, 139°38.3'E) which Guinot (1995: 207, fig. 11b) referred to “Dicranodromiaaff.doederleini”. She described the carapace, proepistome, antennae, antennules, buccal frame, ventral surfaces and merus of the third maxilliped are being more spiny than typical *D.doederleini* even though the outer surface of the chela was smooth. The more spinous features of the specimen (notably the ventral surfaces, antennae, epistome and third maxillipeds), resemble the condition in *D.danielae*, but whether the flexor margin of the pereiopods of the specimen was also spinous was not stated. In addition, the carapace of *D.danielae* is less spinous compared to that figured by Guinot (1995: fig. 11b) for her “Dicranodromiaaff.doederleini”. It would appear that this Japanese specimen is a species close to, but probably different from, *D.danielae*.

#### Dicranodromia erinaceus sp. nov.

Taxon classificationAnimaliaDecapodaHomolodromiidae

4F563B84-BA4F-5460-A96A-9606FBC33594

http://zoobank.org/C1BEA5CC-F350-47D1-8A66-ECBFC3606197

**Figure 13.**
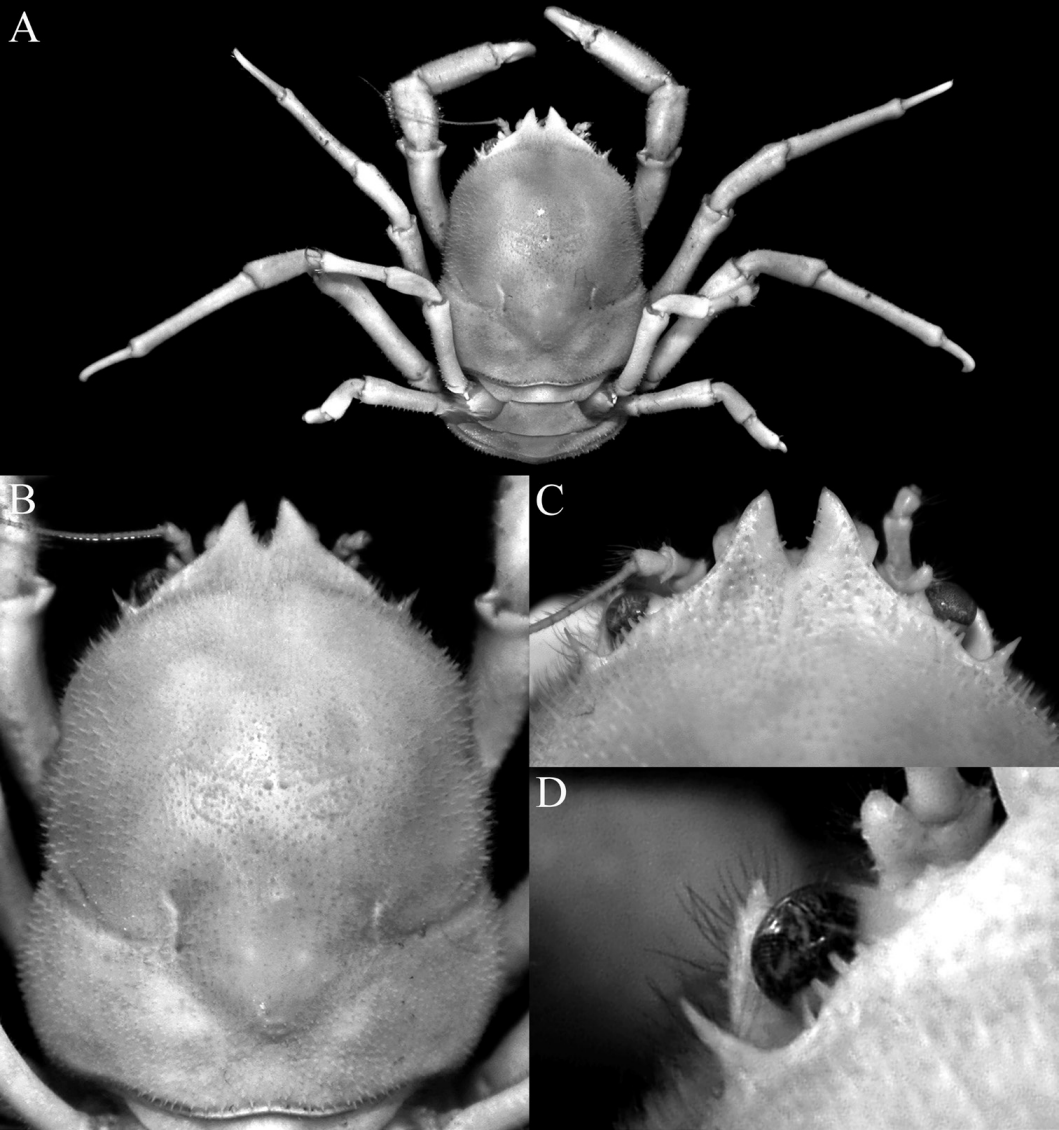
*Dicranodromiaerinaceus* sp. nov., holotype ♀ (14.0 × 18.0 mm) (NTOU B00126), Taiwan **A** overall view **B** dorsal view of carapace **C** front and anterior part of carapace **D** left orbit and first anterolateral spine.

**Figure 14.**
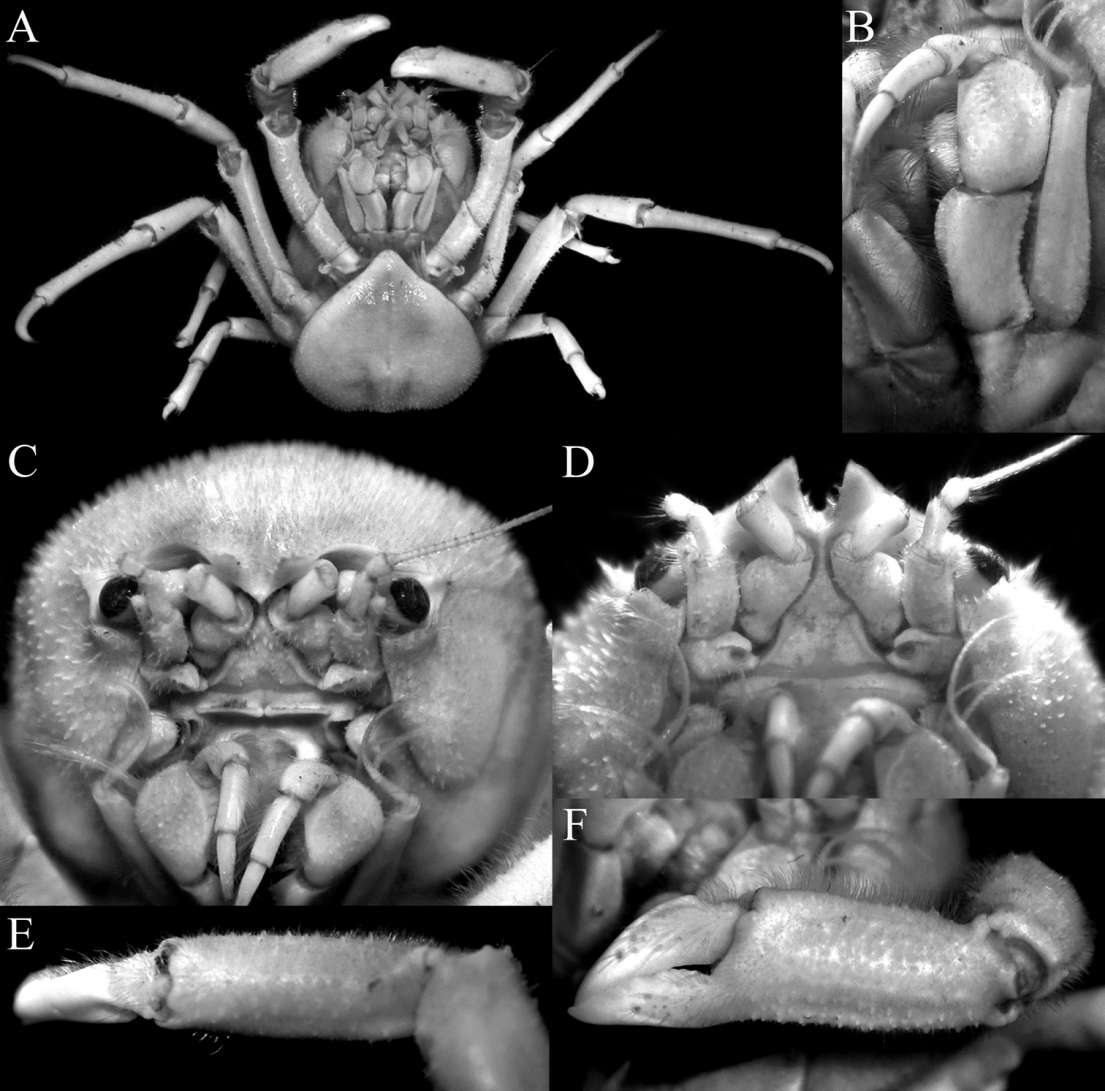
*Dicranodromiaerinaceus* sp. nov., holotype ♀ (14.0 × 18.0 mm) (NTOU B00126), Taiwan **A** ventral view of cephalothorax **B** left third maxilliped **C** frontal view of cephalothorax **D** epistome, antennules, antennae and orbits **E** dorsal view of right chela **F** left chela.

**Figure 15.**
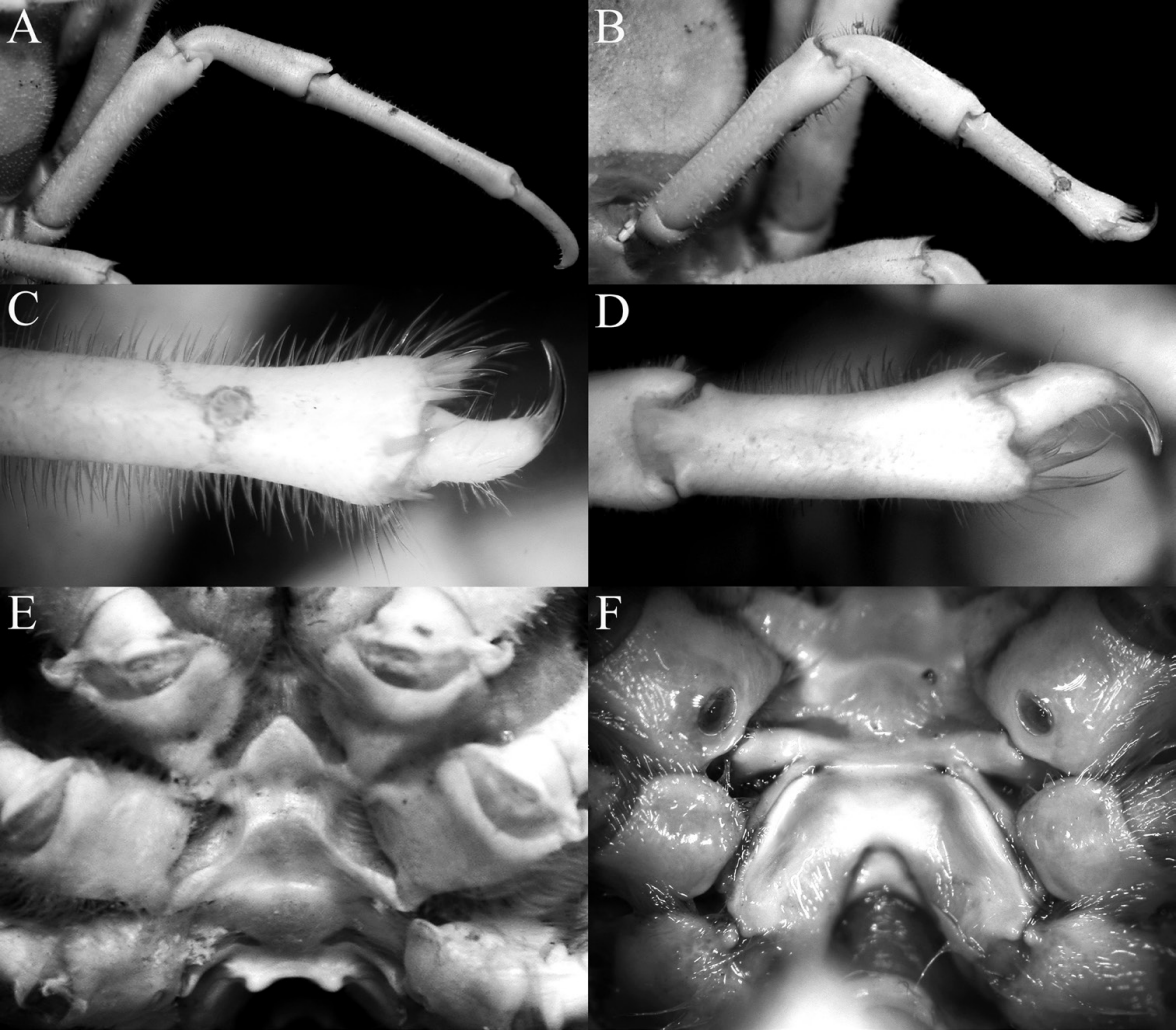
*Dicranodromiaerinaceus* sp. nov. **A–E** holotype ♀ (14.0 × 18.0 mm) (NTOU B00126), Taiwan **F** paratype ♀ (13.8 × 18.5 mm) (ZRC 2020.0467), Taiwan **A** right P3 **B** right P5 **C** right P5 propodus and dactylus **D** right P4 propodus and dactylus **E** anterior thoracic sternum and spermatheca **F** posterior thoracic sternum showing spermatheca and female gonopores.

**Figure 16.**
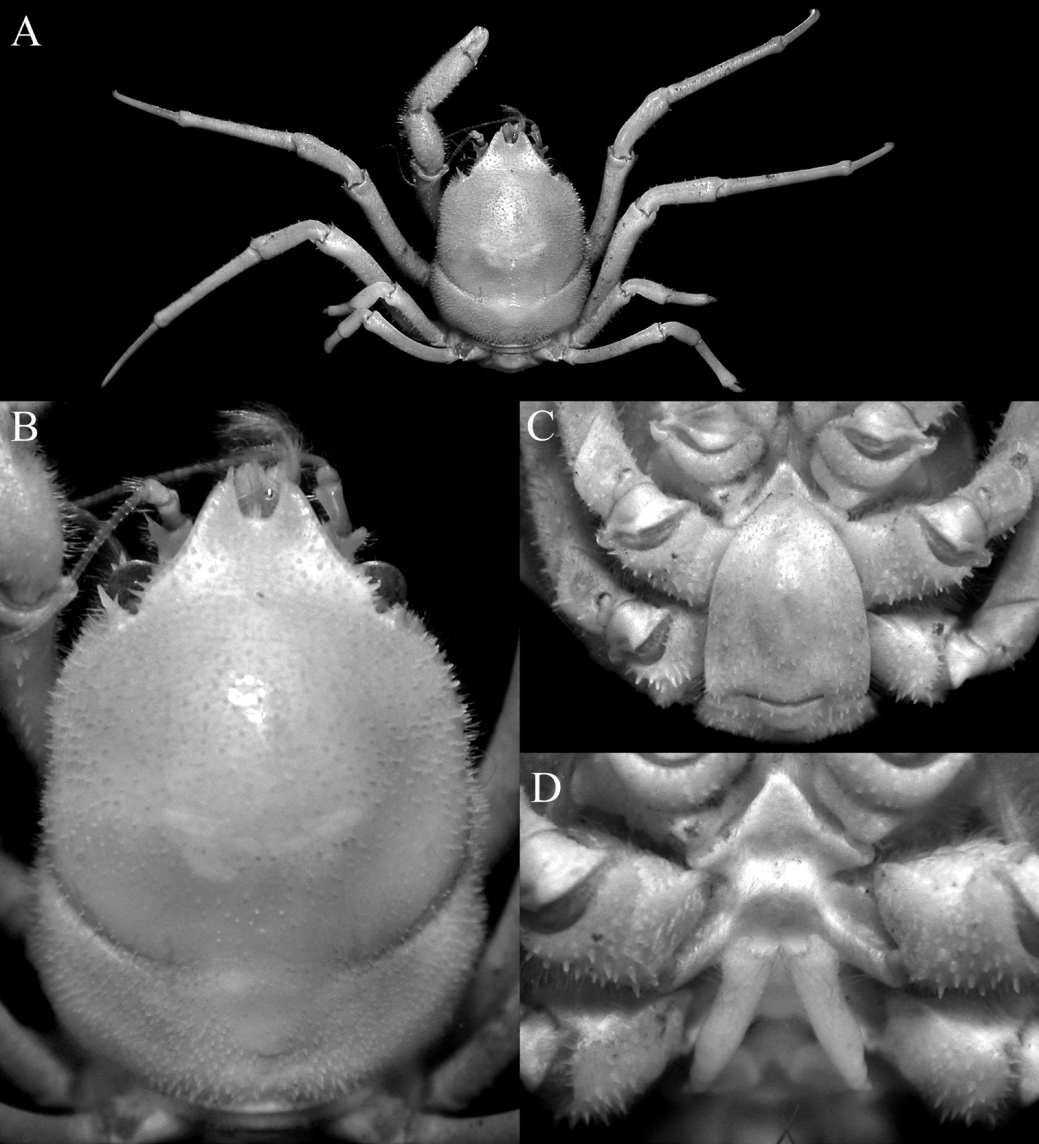
*Dicranodromiaerinaceus* sp. nov., paratype ♂ (8.2 × 12.5 mm) (ZRC 2021.0085), Taiwan **A** overall view **B** dorsal view of carapace **C** pleon **D** anterior thoracic sternum and G1s in situ.

Figures 7E, F , 13 , 14 , 15 , 16 , 21A, B, D–F, K–M

Dicranodromia doederleini – Ho et al. 2004: 643, fig. 1B; Ahyong et al. 2009: 129, fig. 93 (not fig. 94); Ng et al. 2017: 27 (list). (non Dicranodromiadoederleini Ortmann, 1892)

##### Material examined.

TAIWAN: holotype ♀ (14.0 × 18.0 mm), station CP4091, 22°14'N, 119°59'E, among numerous mud tubes, off small Liu-Qiu Island, southeast Taiwan, 974–994 m, coll. N.O. Ocean Researcher 1, 27 May 2013 (NTOU B00126); paratypes 2 ♀♀ (13.2 × 17.6 mm, 13.8 × 18.5 mm), same data as holotype (ZRC 2020.0467, COI sequence: OK351335); 1 ♂ (6.9 × 9.5 mm), station CP4212, 22^°^18.34'N, 119^°^59.51'E, southwestern Taiwan, 961–1008 m, coll. T.-Y. Chan, 15 Nov. 2020 (ZRC 2021.0084, COI sequence: OK331334); 1 ♂ (8.2 × 12.5 mm), station CP4212, 22^°^18.34'N, 119^°^59.51'E, southwestern Taiwan, 961–1008 m, coll. T.-Y. Chan, 15 Nov. 2020 (ZRC 2021.0085). Others: 1 ♀ (7.5 × 11.1 mm, carapace badly damaged), 24°26.9'N, 122°18.1'E, Taiwan, 638–824 m, coll. R/V “Fishery Researcher 1”, 4 August 2000 (NTOU B00127).

##### Diagnosis.

Carapace longitudinally subovate, widest across intestinal-mesobranchial regions; dorsal surface prominently convex, lateral surfaces covered with numerous spinules, those on median part relatively lower, sometimes granular, with short stiff setae, denser on lateral parts but not obscuring margins; short stiff setae present on pereiopods, thoracic sternum and pleon but not obscuring surface or margins. Branchiocardiac groove distinct, curving medially anteriorly. Each pseudorostral lobe triangular, inner margin straight, outer margin gently convex, directed anteriorly, inner margin with two or three spinules; exorbital tooth spiniform, directed obliquely laterally, anterior margin with two or three spinules; supraorbital margin separated from external orbital tooth by shallow concave cleft, posterior part with three spines; infraorbital margin with prominent triangular lobe, posterior margin with spinules, just visible in dorsal view. Rostrum present as one or two longer spinules in small specimens, barely discernible or just visible as a sharp granule in larger specimens. Epistome covered with spinules on anterior half; posterior half gently upturned, with median fissure, surface not covered with spinules, posterior margin entire. Basal antennal article subquadrate; surfaces covered by spinules and granules; anteroexternal tooth short. Eyes with short peduncle. Third maxilliped relatively narrow; merus subovate with low anterointernal lobe, slightly shorter than ischium; ischium subtrapezoidal, distal half wider than proximal part with inner margin convex; palp (carpus, propodus, dactylus) long, reaching to median part of ischium when folded; exopod with proximal third widest, outer margin with low sharp granules on proximal third. Chelipeds densely covered with stiff setae on most parts; merus and carpus with outer surface and margins lined with spinules and granules; palm relatively short, outer surface and margins covered with numerous sharp granules; fingers thick, wide, occluding surface hollowed; pollex with deep U-shaped depression distally. P2 and P3 relatively long, P3 longer than P2; merus with low tooth on distal extensor margin, length to width ratio of P2 and P3 merus 5.2 and 4.5, respectively; proximal part of extensor margin with low spinules, flexor margin with numerous spinules; propodus almost straight, unarmed, length to width ratio of P2 and P3 propodus 6.7 and 8.0, respectively; dactylus sickle-shaped, flexor margin lined with 15 or 16 spines, terminating in strongly incurved claw, propodus about twice length of dactylus. P4 stouter, shorter than P5; length to width ratio of P4 and P5 merus 3.5 and 5.0, respectively; proximal part of extensor margin of merus with low spinules, flexor margin with numerous spinules; P4 and P5 propodus without median spinules on outer surface, length to width ratio of P4 and P5 propodus 3.5 and 4.7, respectively, distal margin fringed by sharp spines bracketing dactylus; dactylus claw-like, strongly incurved, extensor margin unarmed, flexor margin unarmed or with two weak spines. Thoracic sternite 7 with strong transverse ridge from posterior inner part of female gonopore, lateral part raised, forming triangular tubercle, curving posteriorly to join oblique ridge formed by sternites 7 and 8 with distinct groove between them that leads to spermathecal aperture at centre of triangular tubercle. Male and female pleons with six free somites and telson; male telson distinctly subovate; female telson wide, triangular, with gently sinuous margins. G1 stout, endopod distally covered by dense long setae, subdistal part of outer margin with two lobes, proximal lobe larger, prominent; G2 endopod gradually tapering to sharp tip.

##### Variation.

None of the specimens examined had a spine or spinule on the extensor margin of the P5 dactylus; and outer surface of the P5 propodus was also unarmed (Fig. 21E). Most of the flexor margins of the dactylus were not armed with obvious spines or spinules, although two or three stout setae may be present.

##### Etymology.

The species is named after the hedgehog, *Erinaceus*, alluding to the spiny appearance of the carapace and legs. The name is used as a noun in apposition.

##### Remarks.

*Dicranodromiaerinaceus* sp. nov. belongs to the same group of species as *D.spinulosa* and *D.delli* in its spinose carapace surface and pereiopods, slender and spiniform exorbital tooth, and an acutely triangular suborbital tooth. *Dicranodromiaerinaceus* is most similar to *D.delli* from New Zealand but can be distinguished by the ischium of the third maxilliped being relatively shorter and wider especially at the distal half (Fig. 14B) (versus more slender and rectangular in *D.delli*, cf. Ahyong 2008: fig. 4C); proportionately shorter P2 and P3 (e.g., P3 merus 4.5× longer than wide, propodus 8.0× longer than wide, Figs 13A, 15A) (versus P3 merus 6.6× longer than wide, propodus 11.1× longer than wide in *D.delli*, cf. Ahyong 2008: fig. 2A, 3D); the proportionately shorter and stouter P4 and P5 (e.g., P5 merus just reaches the branchiocardiac groove in dorsal position, Figs 13A, 15B) (versus longer and more slender, extending beyond branchiocardiac groove in dorsal position in *D.delli*, cf. Ahyong 2008: fig. 2A, B); the relatively stouter palm (Fig. 14F) (versus more slender in *D.delli*, cf. Ahyong 2008: fig. 3B); and the proportionately wider female telson (Fig. 14A) (versus less wide in *D.delli*, cf. Ahyong 2008: fig. 3C). The holotype and only known specimen of *D.delli*, an ovigerous female 15.5 × 19.0 mm from Nukuliau Seamount in New Zealand, is comparable in size to the ovigerous female holotype of *D.erinaceus* (14.0 × 18.0 mm) so the differences are not size-related. The characters of P2–P5 and third maxilliped are also obvious in the smaller female paratype of *D.erinaceus* as well as in the smaller male paratypes.

Compared to *D.spinulosa*, *D.erinaceus* can be separated by the carapace being proportionately wider (Figs 13B, 16B) (versus carapace transversely narrower in *D.spinulosa*, cf. Guinot 1995: fig. 21a; Ahyong 2008: fig. 1C); the median dorsal surface of the carapace covered with low sharp granules (Figs 13B, 16B) (versus covered with spinules in *D.spinulosa*, cf. Guinot 1995: fig. 21a; Ahyong 2008: fig. 1C); the submarginal surface of the posterior margin of the epistome is unarmed (Fig. 14C, D) (versus area armed with short spines in *D.spinulosa*, cf. Guinot 1995: fig. 22B); the ischium of the third maxilliped relatively shorter and wider especially at the distal half (Fig. 14B) (versus more slender and rectangular in *D.spinulata*, cf. Guinot 1995: fig. 21c); the relatively longer P2 and P3 (e.g., P3 propodus 8.0× longer than wide, Figs 13A, 15A) (versus P3 propodus less than 7× longer than wide), the P2 and P3 propodus twice the length of the dactylus (Fig. 15A) (versus 1.7× in *D.spinulosa*; Guinot 1995: fig. 21a; Ahyong 2008: fig. 1C); and the male telson subovate in shape (Fig. 16C) (versus triangular in *D.spinulosa*, cf. Guinot 1995: figs 21c, 25D). *Dicranodromiaspinulosa* was described from three males and one female from New Caledonia, the holotype female being 7.5 × 11.0 mm; a size comparable to that of the male specimens of *D.erinaceus* we examined.

Ho et al. (2004) recorded *D.doederleini* from Taiwan from one badly damaged female specimen from northeastern Taiwan (see also Ahyong et al. 2009). The specimen is now referred to *D.erinaceus*.

#### Dicranodromia robusta sp. nov.

Taxon classificationAnimaliaDecapodaHomolodromiidae

E5583473-0569-5F91-A871-19A9F9822336

http://zoobank.org/ADCE5085-D836-4A3E-8CF9-5BEB79ED3FE1

**Figure 17.**
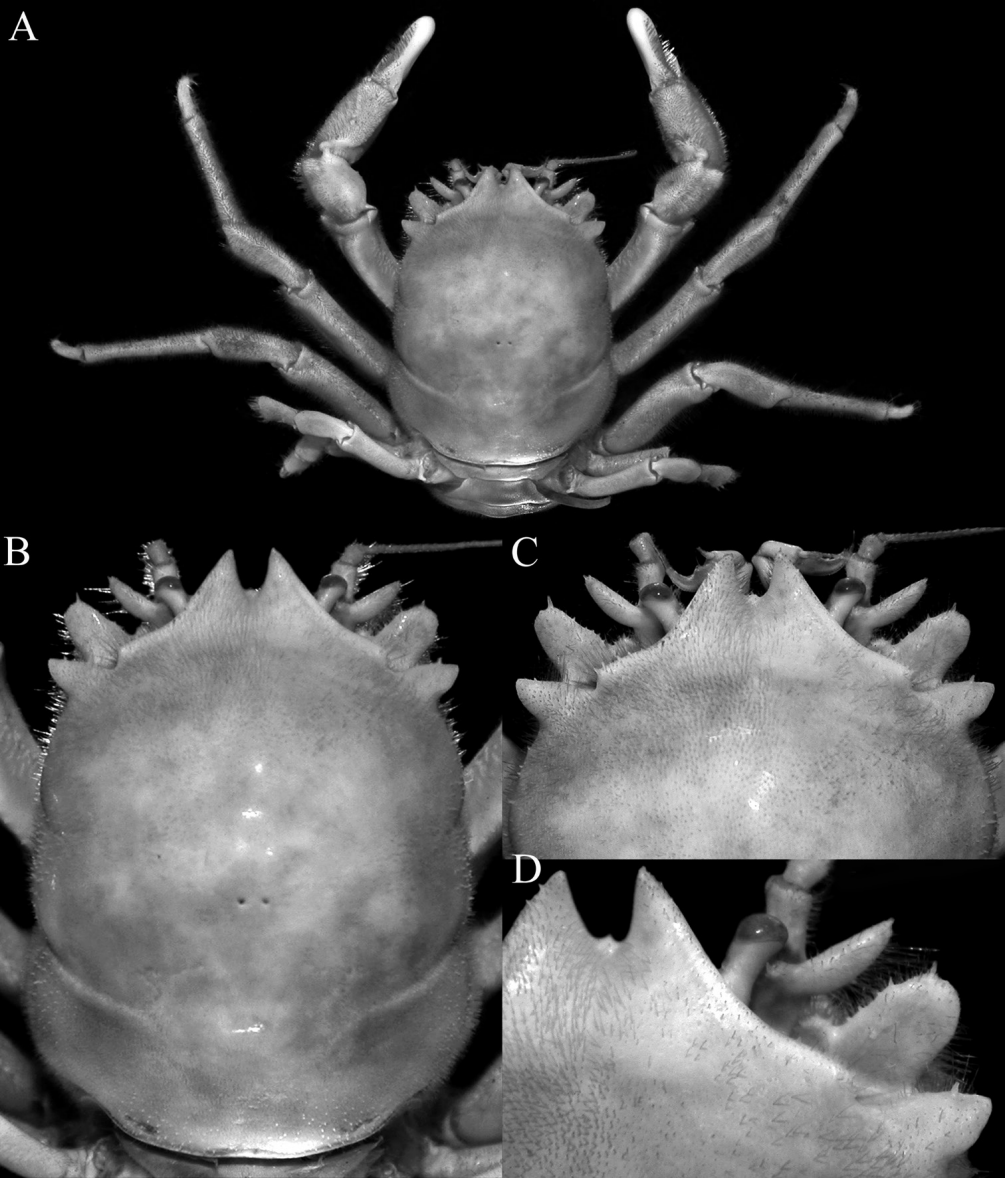
*Dicranodromiarobusta* sp. nov., holotype ♀ (19.6 × 26.4 mm) (ZRC 2018.0161), Philippines **A** overall view **B** dorsal view of carapace **C** front and anterior part of carapace **D** right front, orbit and first anterolateral spine

**Figure 18.**
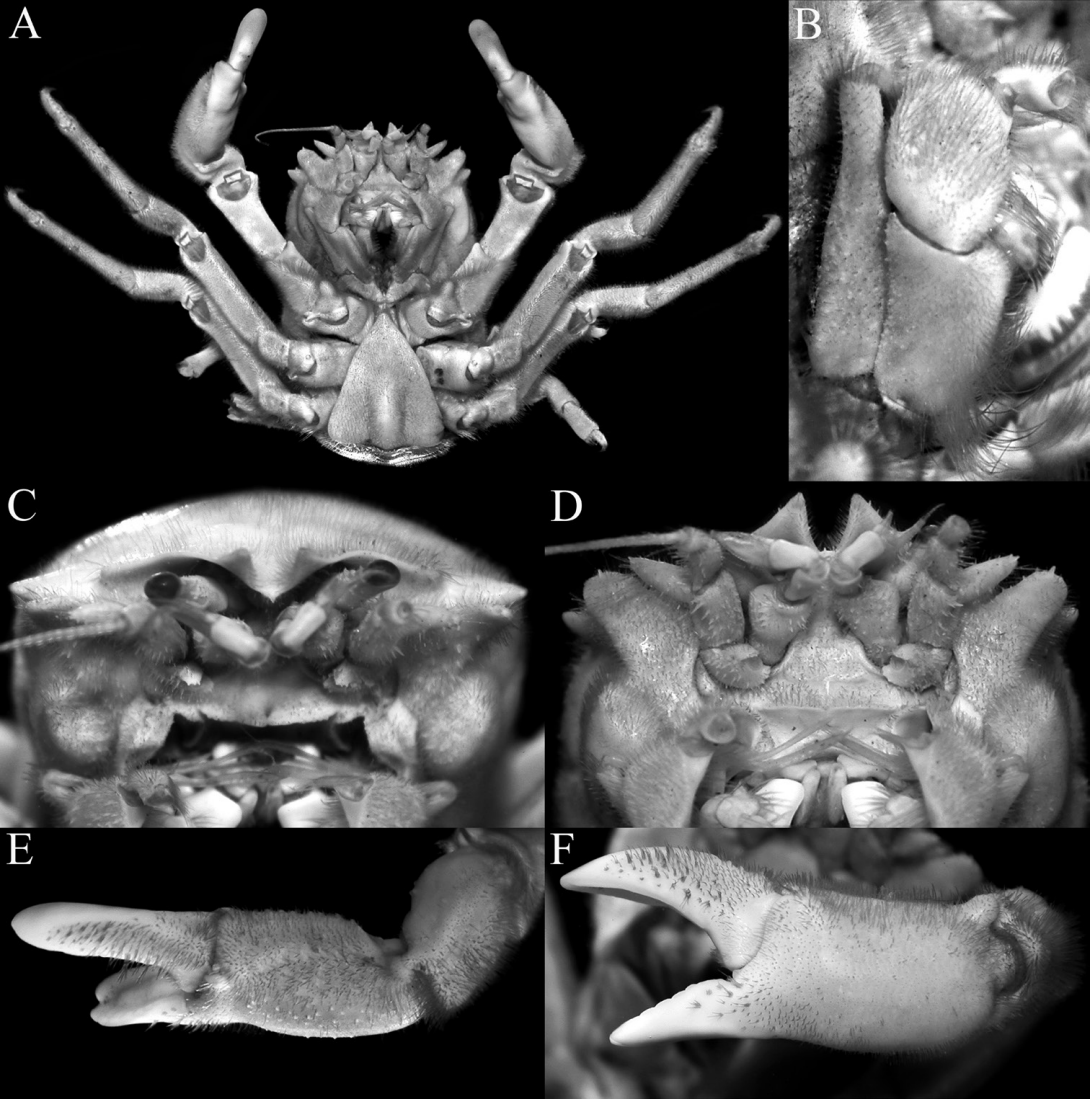
*Dicranodromiarobusta* sp. nov., holotype ♀ (19.6 × 26.4 mm) (ZRC 2018.0161), Philippines **A** ventral view of cephalothorax **B** left third maxilliped **C** frontal view of cephalothorax **D** epistome, antennules, antennae and orbits **E** dorsal view of left chela **F** left chela.

**Figure 19.**
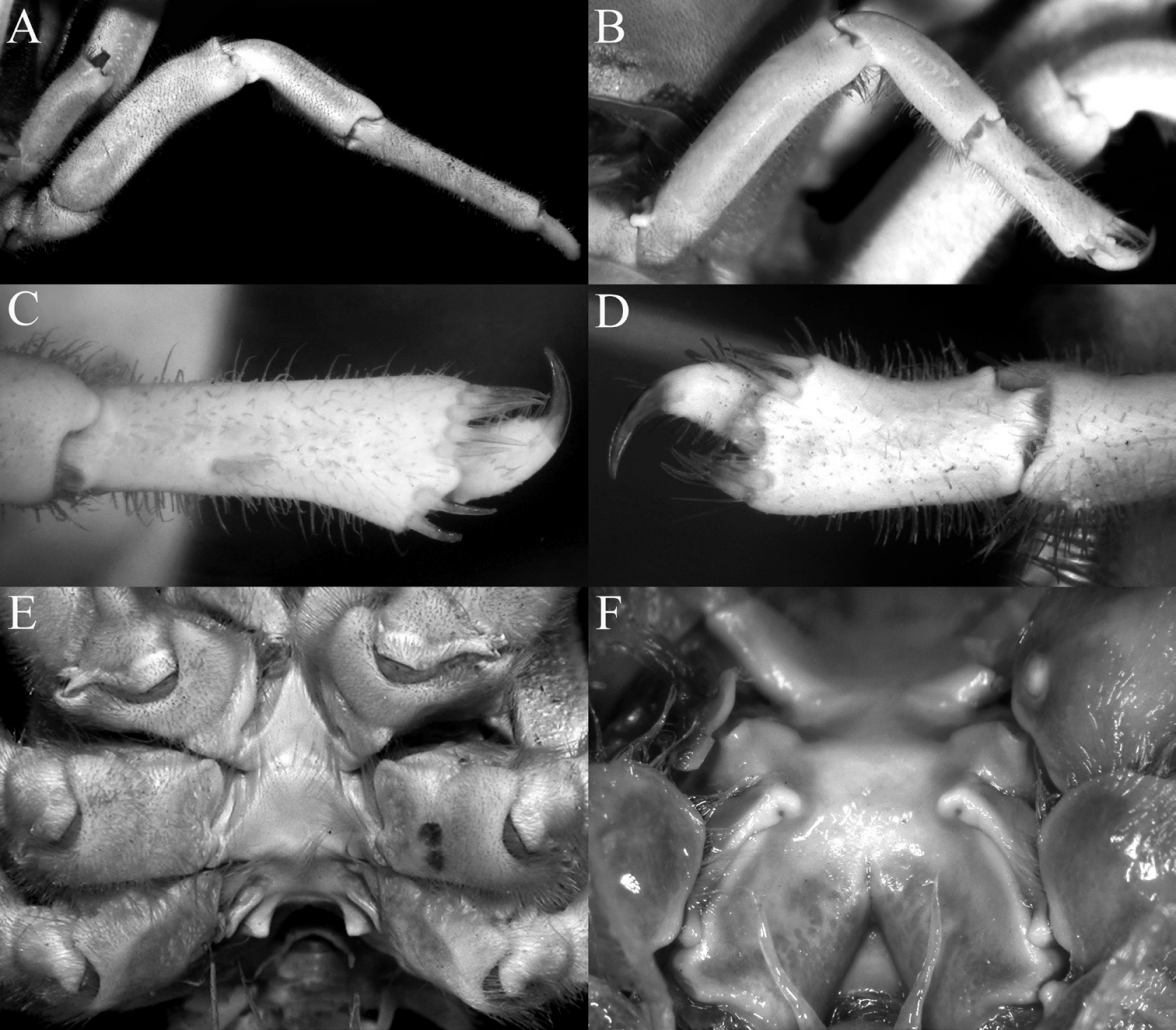
*Dicranodromiarobusta* sp. nov., holotype ♀ (19.6 × 26.4 mm) (ZRC 2018.0161), Philippines **A** right P3 **B** right P5 **C** right P5 propodus and dactylus **D** left P4 propodus and dactylus **E** anterior thoracic sternum and spermatheca **F** posterior thoracic sternum showing spermatheca.

**Figure 20.**
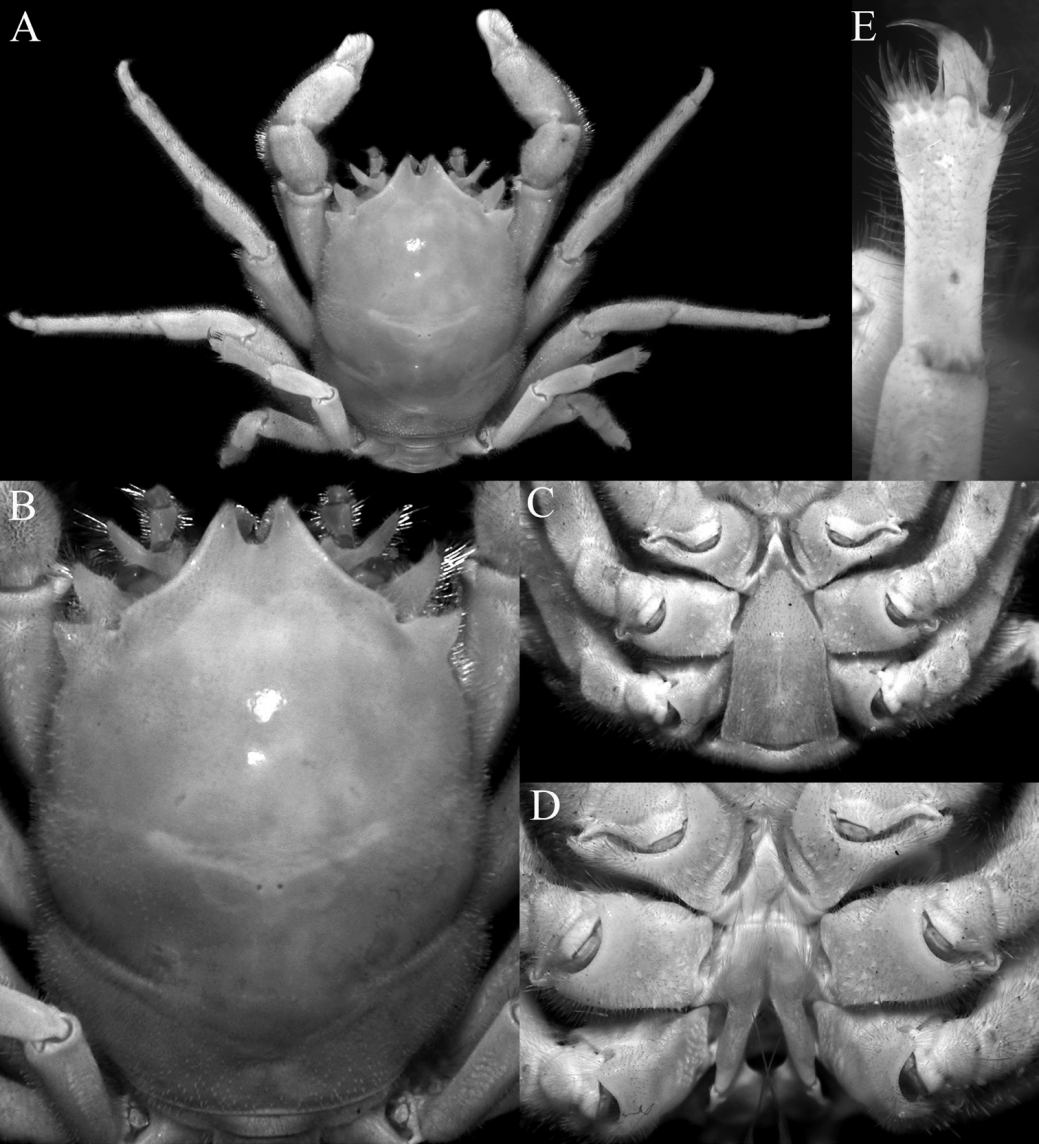
*Dicranodromiarobusta* sp. nov., paratype ♂ (15.2 × 21.0 mm) (ZRC 2018.0095), Philippines **A** overall view **B** dorsal view of carapace **C** pleon **D** anterior thoracic sternum and G1s in situ.

**Figure 21.**
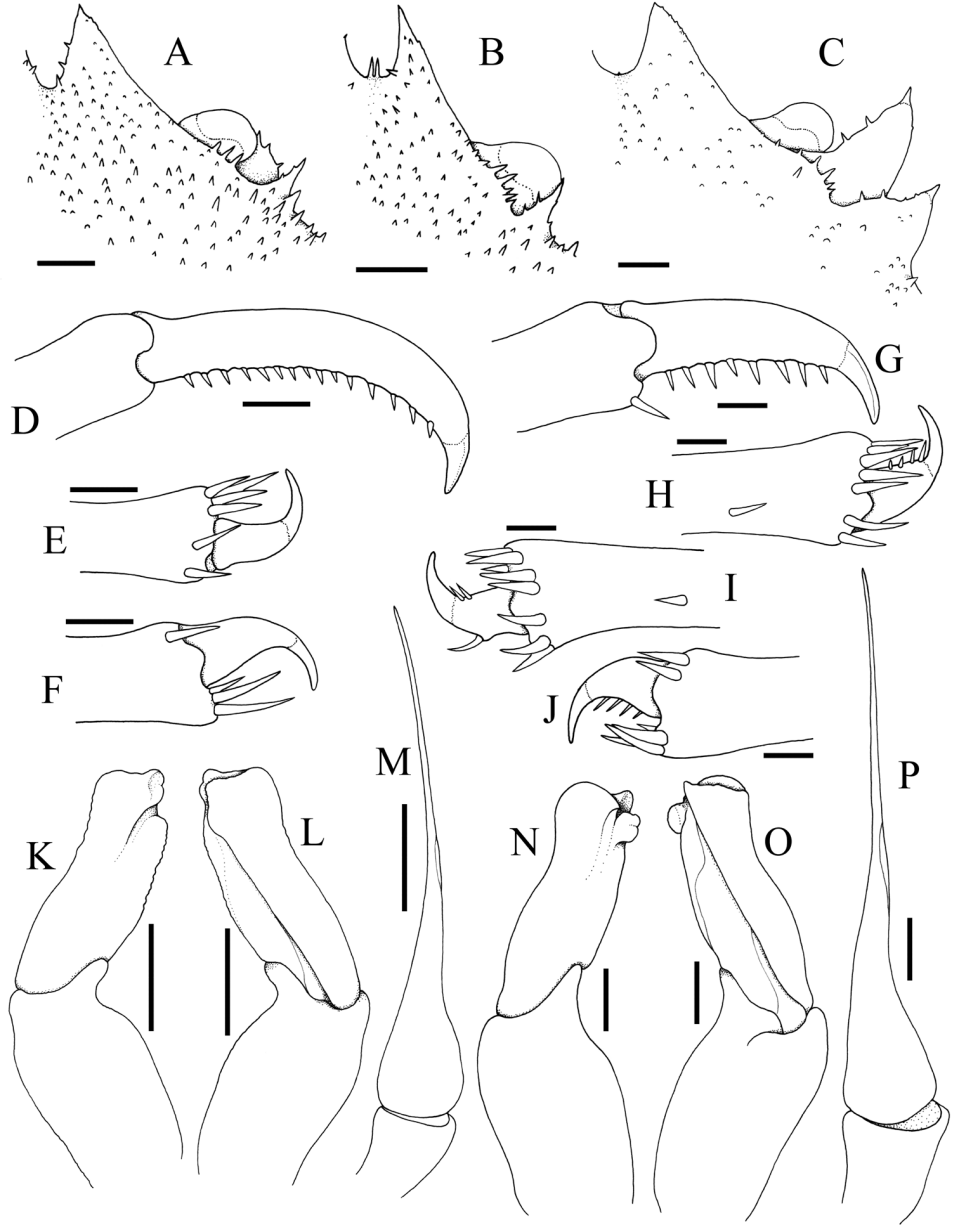
**A***Dicranodromiaerinaceus* sp. nov., holotype ♀ (14.0 × 18.0 mm) (NTOU B00126), Taiwan **B, D–F, K–M***D.erinaceus* sp. nov., paratype ♂ (8.2 × 12.5 mm) (ZRC 2021.0085), Taiwan **C, G–J, N–P***D.robusta* sp. nov., paratype ♂ (15.2 × 21.0 mm) (ZRC 2018.0095), Philippines **A–C** right anterior part of carapace (setae removed or not drawn) **D** right P3 propodus and dactylus **E** right P5 propodus and dactylus **F** right P4 propodus and dactylus **G** right P3 propodus and dactylus **H** right P5 propodus and dactylus **I** left P5 propodus and dactylus **J** left P4 propodus and dactylus **K, N** left G1 (ventral view) **L, O** left G1 (dorsal view) **M, P** left G2. Setae for all structures not figured. Scale bars: 1.0 mm.

Figures 17 , 18 , 19 , 20 , 21C, G–J, N–P

##### Material examined.

Philippines: ***Holotype*** ♀ (19.6 × 26.4 mm), ca. 5°24'N, 125°22.5'E, Balut Island, Sarangani Islands, Davao Occidental Province, south of Mindanao Island, coll. tangle nets, local fishermen, 26 Nov. 2017 (ZRC 2018.0161); ***Paratype*** ♂ (15.2 × 21.0 mm), same location as holotype, coll. tangle nets, local fishermen, 2017 (ZRC 2018.0095).

##### Diagnosis.

Carapace longitudinally subquadrate, widest across intestinal-mesobranchial regions; dorsal surface gently convex, lateral surfaces covered with low spinules, median part smooth, margins with scattered short stiff setae, not obscuring margins; short stiff setae present on pereiopods, thoracic sternum and pleon but not obscuring surface or margins. Branchiocardiac groove distinct, curving medially anteriorly. Each pseudorostral lobe triangular, inner margin straight, outer margin gently convex, directed anteriorly, inner margin entire; exorbital tooth dentiform, directed obliquely laterally, anterior margin with two or three spinules; supraorbital margin separated from external orbital tooth by shallow concave cleft, posterior part with five or six spinules; infraorbital margin with large dorsoventrally flattened lobe which is dentiform to linguiform, larger than exorbital tooth, distal part with spine, anterior margin with two spinules, prominently visible in dorsal view. Rostrum present as one sharp granule. Epistome covered with scattered granules on anterior half; posterior half gently upturned, with median fissure, surface not covered with spinules, posterior margin gently convex, median part entire, lateral part gently serrate. Basal antennal article subquadrate; surfaces covered by spinules and granules; anteroexternal tooth short. Eyes with long peduncle. Third maxilliped relatively narrow; merus subovate with low anterointernal lobe, shorter than ischium; ischium subtrapezoidal, distal half slightly wider than proximal part; palp (carpus, propodus, dactylus) long, reaching to median part of ischium when folded; exopod with proximal third widest. Chelipeds covered with stiff setae on most parts; merus and carpus with margins uneven or lined with granules; palm relatively short, subdorsal and subventral margins with low sharp granules, median part smooth; fingers thick, wide, occluding surface hollowed; pollex with deep U-shaped depression distally. P2 and P3 relatively short, P3 longer than P2; merus with low tooth on distal extensor margin, length to width ratio of P2 and P3 merus 4.2 and 3.9, respectively; margins unarmed; propodus almost straight, unarmed, length to width ratio of P2 and P3 propodus 5.2 and 6.4, respectively; dactylus curved, flexor margin lined with 8 or 9 spines, terminating in strongly gently curved claw, propodus about 2.4× length of dactylus. P4 stouter, shorter than P5; length to width ratio of P4 and P5 merus 2.4 and 3.4, respectively; margins of merus unarmed; P4 and P5 propodus with submedian spinule on distal third of outer surface, length to width ratio of P4 and P5 propodus 2.3 and 3.6, respectively, distal margin fringed by sharp spines bracketing dactylus; dactylus claw-like, strongly incurved, extensor margin with median spine or absent, flexor margin with 2–4 spines. Thoracic sternite 7 with low transverse ridge from posterior inner part of female gonopore, lateral part high, forming triangular tubercle, curving posteriorly to join oblique ridge formed by posterior part of sternite 7, just before suture with sternite 8, groove between sternites 7 and 8 curve to join spermathecal aperture at base of triangular tubercle. Male and female pleons with 6 free somites and telson; male telson distinctly elongate, triangular with gently convex lateral margins; female telson triangular, with gently convex margins. G1 stout, endopod distally covered by dense long setae, subdistal part of outer margin with two lobes, the distal one being more prominent; G2 endopod gradually tapering to sharp tip.

##### Variation.

In the holotype female, the left P5 dactylus has a prominent spine on the extensor margin (Fig. 21I), but there is none on the right side (Fig. 21H). The P5 dactyli of the paratype male are armed a spinule on the extensor margin. Both specimens possess the spine on the outer surface of the P5 propodus (Fig. 21H, I).

##### Etymology.

The species is named after the Latin *robusta* for stout, alluding to the stocky appearance of the species.

##### Remarks.

The most diagnostic character of *D.robusta* sp. nov. is the large dorsoventrally flattened infraorbital tooth, which is dentiform to linguiform, clearly visible in dorsal view, and distinctly larger than the exorbital tooth (Figs 17B–D, 20B). No other Indo-West Pacific species of *Dicranodromia* has such a large and wide infraorbital tooth. The long anteroexternal tooth on the basal antennal article allies *D.robusta* with *D.martini* (cf. Guinot 1995: fig. 20B), *D.baffini* (cf. Alcock 1899: pl. 2 fig. 1a; Alcock 1901: pl. 1 fig. 1a), *D.danielae* (cf. Ng and McLay 2005: fig. 3A, B) and *D.chenae* (cf. Ng and Naruse 2007: fig. 5b) but the structure of the infraorbital tooth easily distinguishes it from them.

The carapace shape of *D.robusta* is distinctly more quadrate (Figs 17A, B, 20A, B) than the more pyriform *D.martini* described from the Philippines (cf. Figs 4A, C, 6A, B; Guinot 1995: fig. 19b; Ng and Naruse 2007: fig. 1a–c); the posterior margin of the epistome is entire (Fig. 18C) (versus margin gently crenulate in subventral view in *D.martini*, cf. Guinot 1995: fig. 20B); P2 and P3 are prominently shorter with the dactylus especially short (e.g., P3 merus 3.9× longer than broad, propodus 6.4× longer than broad, Fig. 19A) (versus P3 merus 7.0× longer than broad, propodus 7.2× longer than broad in *D.martini*, cf. Figs 4A, 5C, 6A; Guinot 1995: figs 19a, e, 20C); P4 and P5 are much shorter (e.g., P4 merus just reaching branchiocardiac groove when folded dorsally, Figs 17A, 19B, 20A) (versus P4 merus long, reaching beyond branchiocardiac groove when folded dorsally in *D.martini*, cf. Figs 4A, 5D, 6A; Guinot 1995: fig. 19a); and the male telson is lingulate (Fig. 20C) (versus more elongate in *D.martini*, cf. Fig. 6D; Guinot 1995: fig. 19c).

Compared to *D.baffini* from the Indian Ocean, *D.robusta* has a more quadrate carapace (Figs 17A, B, 20A, B) (versus more pyriform in *D.baffini*, cf. Alcock 1899: pl. 2 fig. 1a; Guinot 1995: fig. 13; Padate et al. 2020: fig. 2a); and the P2 and P3 dactylus is distinctly shorter (Figs 19A, 21G) (versus longer in *D.baffini*, cf. Alcock 1899: pl. 2 fig. 1a; Guinot 1995: fig. 13). With regards to the relatively shorter P2 and P3 dactyli, *D.robusta* resembles *D.chenae*, described from a single large ovigerous female from the central Philippines. *Dicranodromiarobusta*, however, can easily be distinguished in having the outer margin of the pseudorostral lobe is almost straight and the structure is directed anteriorly (Figs 17B–D, 20B) (versus outer margin of the pseudorostral lobe is distinctly convex with the structure gradually curved inwards towards the median in *D.chenae*, cf. Ng and Naruse 2007: fig. 5A); the ischium of the third maxilliped is short and rectangular (Fig. 18B) (versus distinctly longer and more slender in *D.chenae*, cf. Ng and Naruse 2007: fig. 5b); the female telson is relatively more elongate (Fig. 18A) (versus proportionately wider and shorter in *D.chenae*, cf. Ng and Naruse 2007: fig. 2b); and the spermatheca is on the prominently raised part around the suture between sternites 7 and 8 and ends at the centre of the triangular tubercle on sternite 6 (Fig. 19F) (versus spermatheca is not prominently raised and ends at the base of the triangular tubercle in *D.chenae*, cf. Ng and Naruse 2007: fig. 8).

*Dicranodromiarobusta* can be separated from *D.danielae* in having the exorbital tooth distinctly triangular to linguiform (Figs 17B–D, 21C) (versus subtrapezoidal in *D.danielae*, cf. Fig. 12A, B; Ng and McLay 2005: figs 1B, 4A); the posterior margin of the epistome is entire (Fig. 18C) (versus clearly serrate in *D.danielae*, cf. Fig. 12C; Ng and McLay 2005: fig. 4C); the median part of the outer surface of the chela is granular (Fig. 18E, F) (versus smooth in *D.danielae*, cf. Fig. 12D; Ng and McLay 2005: fig. 3A, B); and P2–P5 are all proportionately longer with the flexor margins of the meri not spinate (Figs 17A, 19A, 20A) (versus relatively shorter in *D.danielae* with the meri of P2 and P3 distinctly spinate, cf. Fig. 12E, F; Ng and McLay 2005: fig. 1A).

## Discussion

Ng and Naruse (2007) discussed the value of the spine present on the extensor margin of P5 dactylus as a taxonomic character. They noted that it was sometimes present in *D.martini* (cf. comparative material examined above; Ng and Naruse 2007: fig. 3a, b) and was present on the holotype of *D.danielae* (cf. Ng and Naruse 2007: fig. 3c; Fig. 12F, G). In the specimens of *D.doederleini* examined, none of the P5 dactyli possess this spine (Fig. 3D, E). The presence and absence of this spine must therefore be used with caution. The setae on the P5 propodus are a mixture of setae and spines, but there are some setae which are intermediate in proportions, suggesting that the setae and spines are homologous structures, the “spines” on the distal edge and the outer surface of the P5 propodus, and “spines” on the flexor margin of the dactylus are almost certainly derived from the setae. They all have a clearly defined base and articulate with the cuticle. Normal spines and granules are part of the cuticle and there is no defined base. That being said, the extensor margin of the P5 dactylus in all the specimens of *D.doederleini* and *D.erinaceus* sp. nov. we examined are unarmed (Figs 3D, E, 15B, C). In the case of *D.karubar*, some of the P5 dactyli have spines while others do not (Figs 9G, 10D). For *D.robusta* sp. nov., the dactylar spine on the extensor margin is present in both specimens (but missing on the right leg in the holotype), and in addition, there is a prominent spine on the median outer surface of the P5 propodus which is always present (Fig. 21H, I). In *D.doederleini*, the P5 propodus has two or three spines on the outer surface (Fig. 3D, E); there are two spines in *D.martini*, with one or two spines in *D.karubar* (Fig. 9G), while in *D.erinaceus* sp. nov., the P5 propodus is unarmed (Fig. 15B, C).

The armature of the posterior margin of the epistome is a useful character but must be used carefully as well. In species like *D.danielae*, the margin is prominently spinose even when viewed frontally, with spines appearing more prominent when the margin is viewed subventrally (Fig. 12C). In *D.doederleini* and *D.karubar*, the posterior margin is almost entire or only weakly crenulate when viewed frontally or subventrally (Figs 2D, 8D, 9A). In *D.martini*, the margin appears almost entire in frontal view (Fig. 4E) but when viewed subventrally, it is weakly crenulated and uneven, as figured by Guinot (1995: fig. 20B). The structure of the proepistome, present in all the species examined, is relatively conservative, being separated from the epistome only by the lateral clefts, and for all the species, it is triangular in shape and slightly “sunken” into the distal margin of the epistome. In most species, the surface of the proepistome is covered with low granules and setae (Figs 2E, 5A, 9A, 14D, 18D); but in *D.danielae*, the lateral parts have long spinules and the surface also has short spinules (Fig. 12C).

Guinot (1995: fig. 2C) noted that the actual rostrum of *Dicranodromia*, when visible, is present only as a small median tooth or spinule between the two pseudorostral teeth. It must be noted that this character is probably variable to some degree. In *D.martini*, there is no trace of a rostrum (Figs 4D, 6C). In *D.doederleini*, the rostrum is a distinct sharp granule (Fig. 2B; Guinot 1995: fig. 12B). When it is present as a spinule, the structure may be small, brittle and can easily be broken off. In one of the paratype males of *D.erinaceus* sp. nov. (ZRC 2021.0085), the rostrum is composed of three small, slender spinules, which are very minute and delicate (Fig. 21B). The rostral spinules are not clearly visible on the large female specimens of *D.erinaceus* sp. nov. but it may simply have been lost. In *D.danielae*, there are three spinules (Fig. 12B). In *D.robusta* sp. nov., the rostrum is just a sharp but relatively low granule that is barely discernible (Figs 17C, D, 21C), as in the case of *D.karubar* (Fig. 8D). As such, this character should not be relied on to separate taxa.

In general, all species have spinules on some part of the carapace and these are often surrounded by stiff setae which partially obscure the spinules. Cleaning must be done with great care as the spinules (and even some of the spines) are brittle and break easily.

The structures of the G1 and G2 have not been used to separate species and Guinot (1995) only figured them for one American species (*D.maheuxii*). The G1 endopod is the main character and, while they all have a similar shape, the relative proportions differ and the subdistal part of the outer margin has two lobes of differing shapes and sizes. The G1 of the four species examined here show that there are differences between some taxa and can be used as a taxonomic character. The most distinctive is the G1 of *D.karubar*, in which the subdistal lobe on the outer margin of the endopod is curved and beak-like (Fig. 11D, E), distinct from the more rounded structure of its most similar species, *D.baffini*. The G1 endopods of *D.martini* and *D.karubar* (Fig. 11A, B, D, E) are also proportionately longer than those of *D.erinaceus* sp. nov. and *D.robusta* sp. nov. (Fig. 21K, L, N, O). In addition, the subdistal lobe on the outer margin of the G1 endopod in *D.robusta* (Fig. 21N, O) is distinctly more pronounced than either *D.martini* or *D.erinaceus* sp. nov. (Figs 11A, B, 21K, L). As such, G1 structures for *Dicranodromia* species should be described and figured as part of species descriptions.

Guinot (1995: 182) placed more emphasis on the structure of the spermathecal apertures and associated structures on thoracic sternites 7 and 8, pointing that there are clear differences between species. One of the characteristic features is that all the species have a pair of enlarged tubercles on each side of thoracic sternite 7 which are anterior or adjacent to the spermatheca. When viewed frontally, they appear as a pair of rounded or triangular tubercles (Figs 3G, 5H, 9J, 15E, 19F). In some species like *D.doederleini*, *D.martini*, *D.karubar* and *D.robusta* sp. nov., the two tubercles are separate (Figs 3G, 5H, 9J) but in *D.erinaceus* sp. nov., the two tubercles are connected by a clear ridge that bridges them (Fig. 15E). In *D.doederleini*, the tubercle is distinctively curved laterally outwards (Fig. 3G). In two species, *D.baffini* and *D.karubar*, the tubercle is distinctively comma-shaped, with the spermatheca positioned posteriorly to it (Fig. 9J). The suture between sternites 7 and 8, which joins the spermatheca is also differently structured. In most species, the suture is level with the rest of the sternal surface (Figs 3H, 5H, 9J, 19F). In most species, the spermatheca is posterior to the sternal tubercle (Figs 3H, 5H, 19F). In one species, *D.erinaceus* sp. nov., however, the suture is distinctly raised, on a prominent ridge and joins the spermatheca laterally (Fig. 15F).

**Figure 22.**
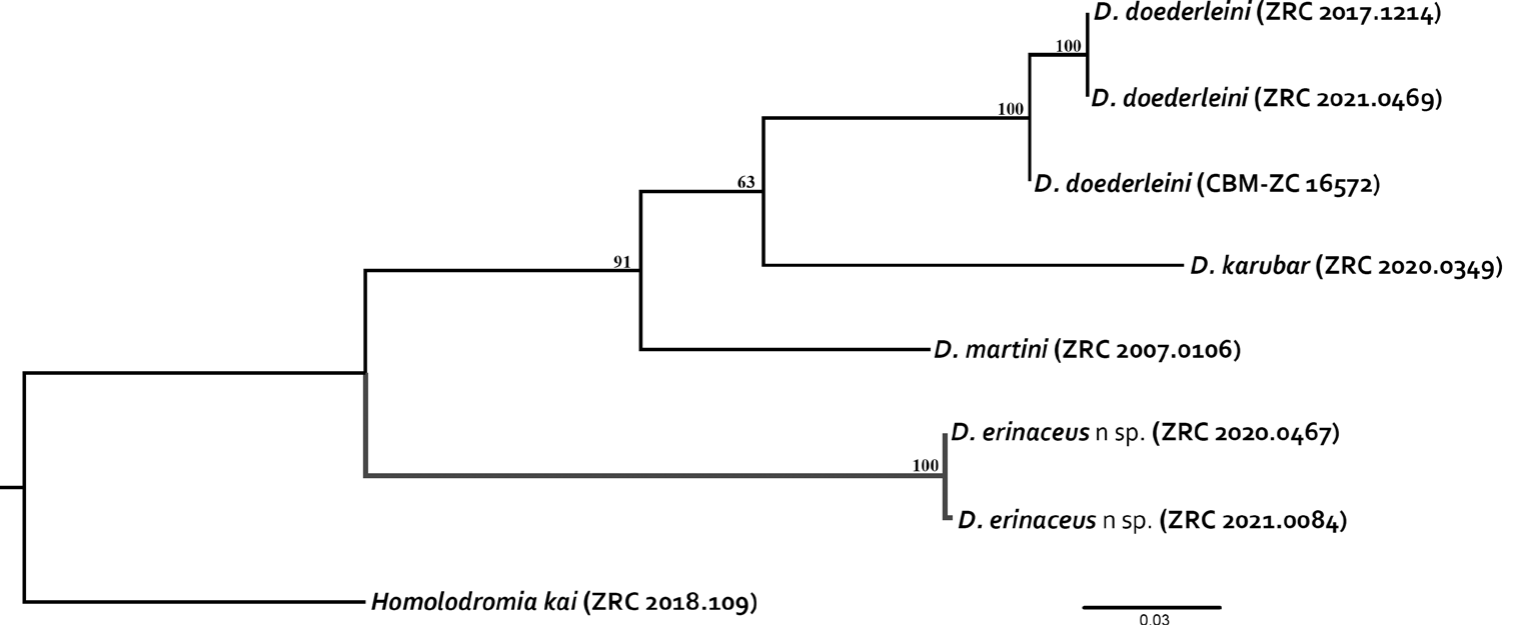
Maximum likelihood phylogenetic tree for *Dicranodromiaerinaceus* sp. nov. based on the COI gene dataset. *Homolodromiakai* Guinot, 1993 was chosen as outgroup. Maximum likelihood bootstrap value is represented as above the branches. Values less than 50 are not shown.

The molecular analyses using COI sequences closely supported the morphological observations. There was some intraspecific divergence in the three individuals of *D.doederleini* tested (GenBank accession nos. OK351331-OK351333), ranging from 0–1.2%, while that in two specimens of *D.erinaceus* sp. nov. (accession nos. OK351334-OK351335) was 0.2%. In *Dicranodromia*, the divergence at the species level was 9.6–14.0%, with *D.erinaceus* sp. nov. distinct from the tested species by 12.8–14.0%. The outgroup *H.kai* (accession no. OK351338) has a minimal divergence of 12.6–12.8% with *D.erinaceus* sp. nov., and a maximal value of 14.2% with *D.karubar*. The maximum likelihood tree also showed *D.erinaceus* sp. nov. to form an independent clade from other *Dicranodromia* species with an extremely high support (MLb = 100) (Fig. 22). The significance of this divergence will need to be re-appraised when more species of *Dicranodromia* (especially the American taxa) can be tested to see if the genus is monophyletic.

Noteworthy is that the Philippines has four species: *D.danielae*, *D.chenae*, *D.martini* and *D.robusta* sp. nov. Two of the species (*D.chenae* and *D.martini*) were collected by trawls, the substrate being more level and less rocky. Like *D.danielae*, *D.robusta* sp. nov. was collected by tangle nets set in deep-water, which may explain why it has not been collected until now. Deep-water habitats with steep rocky substrates are difficult to sample, and the fauna is often different from those occurring in flatter substrates (see Ng et al. 2009; Mendoza et al. 2010). Several other brachyuran taxa show the same pattern, notably in Majoidea (e.g., see Ng and Richer de Forges 2015; Richer de Forges and Ng 2008; Richer de Forges et al. 2021).

## Supplementary Material

XML Treatment for Dicranodromia

XML Treatment for Dicranodromia doederleini

XML Treatment for Dicranodromia martini

XML Treatment for Dicranodromia karubar

XML Treatment for Dicranodromia danielae

XML Treatment for Dicranodromia erinaceus

XML Treatment for Dicranodromia robusta
